# Metabolites with Anti-Inflammatory Activity from the Mangrove Endophytic Fungus *Diaporthe* sp. QYM12

**DOI:** 10.3390/md19020056

**Published:** 2021-01-24

**Authors:** Yan Chen, Ge Zou, Wencong Yang, Yingying Zhao, Qi Tan, Lin Chen, Jinmei Wang, Changyang Ma, Wenyi Kang, Zhigang She

**Affiliations:** 1 National R & D Center for Edible Fungus Processing Technology, Henan University, Kaifeng 475004, China; chenyan27@mail2.sysu.edu.cn (Y.C.); zhaoyingying@vip.henu.edu.cn (Y.Z.); wangjinmei@henu.edu.cn (J.W.); macaya1024@vip.henu.edu.cn (C.M.); 2 School of Chemistry, Sun Yat-Sen University, Guangzhou 510275, China; zoug5@mail2.sysu.edu.cn (G.Z.); yangwc6@mail2.sysu.edu.cn (W.Y.); tanq27@mail2.sysu.edu.cn (Q.T.); 3 Henan Joint International Research Laboratory of Drug Discovery of Small Molecules, Zhengzhou Key Laboratory of Synthetic Biology of Natural Products, Huanghe Science and Technology College, Zhengzhou 450063, China; lchenchina@hhstu.edu.cn

**Keywords:** mangrove endophytic fungus, *Diaporthe* sp., anti-inflammatory activity

## Abstract

One new diterpenoid, diaporpenoid A (**1**), two new sesquiterpenoids, diaporpenoids B–C (**2**,**3**) and three new *α*-pyrone derivatives, diaporpyrones A–C (**4**–**6**) were isolated from an MeOH extract obtained from cultures of the mangrove endophytic fungus *Diaporthe* sp. QYM12. Their structures were elucidated by extensive analysis of spectroscopic data. The absolute configurations were determined by electronic circular dichroism (ECD) calculations and a comparison of the specific rotation. Compound **1** had an unusual 5/10/5-fused tricyclic ring system. Compounds **1** and **4** showed potent anti-inflammatory activities by inhibiting the production of nitric oxide (NO) in lipopolysaccharide (LPS)-induced RAW264.7 cells with IC_50_ values of 21.5 and 12.5 μM, respectively.

## 1. Introduction

**Figure 1:**
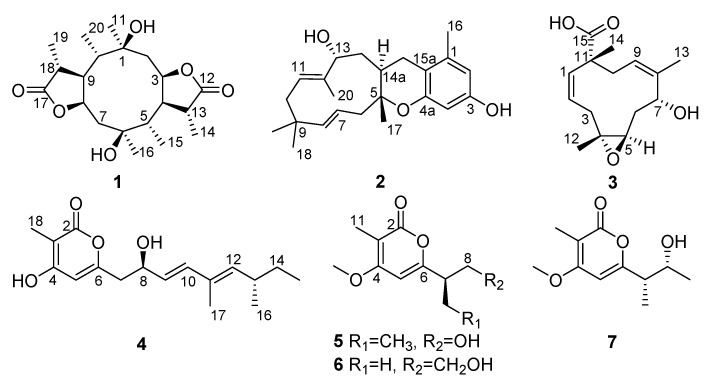
The structures of **1**–**7**.

Mangrove endophytic fungi are the second largest ecological group of the marine fungi [1]. The particular environmental conditions of mangroves allow the activation of unique metabolic pathways in endophytic fungi, enabling the production of novel chemical backbones with diverse biological activities, making them a promising source of drug leads [2,3,4,5]. *Diaporthe* is a ubiquitous fungus commonly isolated from most plant hosts [6]. It is known to produce diverse compounds with antibacterial [7], antifungal [6], cytotoxic [8], antitubercular [9], antiparasitic [10] and anticancer [11] activities. With the aim of seeking new bioactive natural products from marine microorganisms, a mangrove endophytic fungus *Diaporthe* sp. QYM12, which was isolated from *Kandelia candel* collected from the South China Sea, was cultured in solid rice medium. As a result, six new metabolites including diaporpenoids A–C (**1**–**3**) and diaporpyrones A–C (**4**–**6**) together with one known analogue, 4-*O*-methylgermicidin L (**7**) [12], were isolated (Figure 1). Herein, the isolation, structure elucidation and anti-inflammatory activity of all isolated compounds are described.

## 2. Results

**Figure 2:**
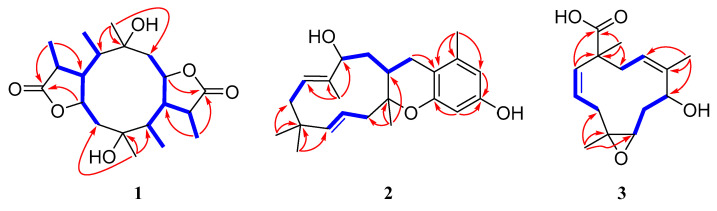
Key HMBC (red arrows) and COSY (blue bold lines) correlations of **1**–**3.**

**Figure 3:**
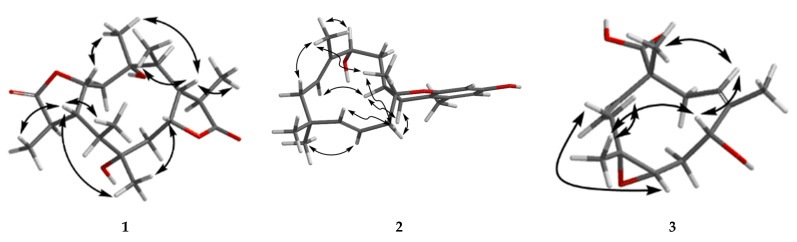
NOESY correlations of **1**–**3.**

**Figure 4:**
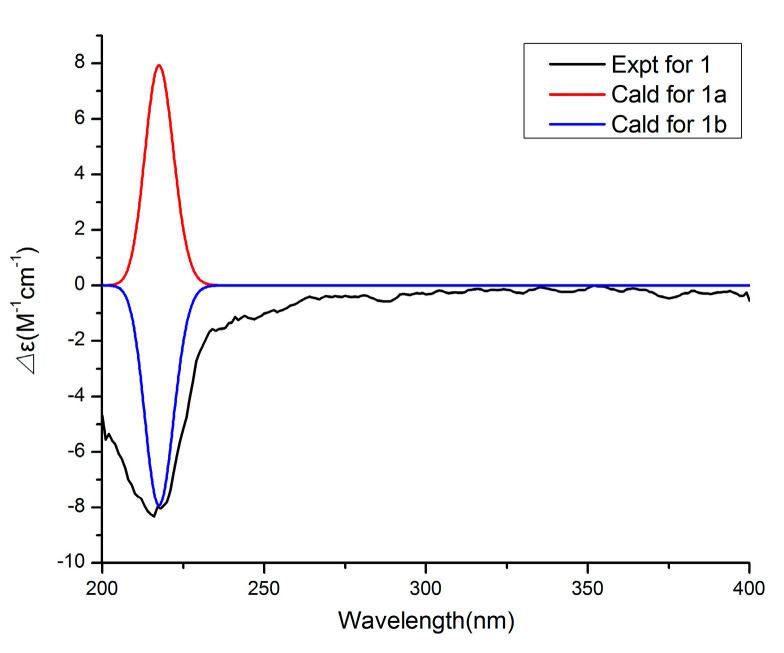
Comparison of the experimental and calculated ECD spectra of **1**.

Compound **1** has a molecular formula of C_20_H_32_O_6_ based on the (+)-HRESIMS (*m*/*z*: 391.20900 [M + Na]^+^), requiring five indices of hydrogen deficiency. The ^1^H NMR data (Table 1) showed six methyl signals at *δ*_H_ 1.20 (s, H_3_-11), 1.33 (d, *J* = 7.6 Hz, H_3_-14), 0.97 (d, *J* = 7.3 Hz, H_3_-15), 1.22 (s, H_3_-16), 1.31 (d, *J* = 7.3 Hz, H_3_-19) and 0.99 (d, *J* = 7.2 Hz, H_3_-20). Twenty carbon resonances in the ^13^C NMR data showed six methyls, two sp^3^ methylenes, eight sp^3^ methines and four quaternary carbons (two carbonyl carbons). These data suggested that **1** may be a tricyclic diterpenoid. The ^1^H-^1^H COSY spectrum revealed two spin systems: H_2_-2/H-3/H-4(/H-5)/H-13/H_3_-14 and H_2_-7/H-8/H-9(/H-10)/H-18/H_3_-19. The HMBC correlations (Figure 2) from H_3_-11 to C-1, C-2 and C-10, and from H_3_-16 to C-5, C-6 and C-7 implied the existence of a ten-membered ring core structure. Moreover, the correlations from H-3 to C-12, from H_3_-14 to C-4 and C-12, from H_3_-19 to C-9 and C-17, and from H-8 to C-17 were consistent with the existence of two five-membered lactones. The NOESY correlations (Figure 3) from H_3_-11/ H-3, H_3_-11/ H-9, H-9/H_3_-20, H-9/H_3_-19, H-4/ H_3_-16, H-4/ H_3_-15, H-4/ H_3_-14 and H_3_-16/H-8 suggested that these protons were cofacial. Thus, the relative configuration of **1** has two possible enantiomers: **1a** (1*R*, 3*S*, 4*S*, 5*R*, 6*R*, 8*S*, 9*S*, 10*R*, 13*S*, 18*S*) and **1b** (1*S*, 3*R*, 4*R*, 5*S*, 6*S*, 8*R*, 9*R*, 10*S*, 13*R*, 18*R*). Comparing the experimental and calculated ECD spectra (Figure 4) between **1** and **1b** at the level of B3LYP/DGDZVP determined the absolution configuration of **1** as 1*S*, 3*R*, 4*R*, 5*S*, 6*S*, 8*R*, 9*R*, 10*S*, 13*R*, 18*R.*

Compound **2** was isolated as a colorless oil and had a molecular formula of C_23_H_32_O_3_ via HRESIMS. The NMR data of **2** were similar to those of pughiinin A [13]. It was confirmed that **2** had the same planar structure as pughiinin A by analyzing the COSY and HMBC correlations (Figure 2). The main difference was the 11*E*-configuration of the double bond between C-11 and C-12, which was confirmed by the NOESY correlation (Figure 3) from Hα-10/H_3_-20. The chemical shift at C-20 (*δ*_C_ 10.6) in **2** further supported the 11*E*-configuration [14]. The relative configuration of **2** was elucidated by the NOESY correlations from H-13/H_3_-20, H_3_-20/H_3_-17, H_3_-17/H-6*β*, H-6*α*/H-15*α* and H-15*α*/H-14a. Thus, the structure of **2** was defined as shown in Figure 1.

The HRESIMS data of **3** suggested a molecular formula of C_15_H_22_O_4_. The ^13^C NMR data (Table 2) showed 15 carbon resonances, including three methyls, three sp^3^ methylenes, five methines (two oxygen-bearing and three olefinic) and four quaternary carbons (one olefinic and one carbonyl). The COSY correlations (Figure 2) revealed the presence of three spin systems from H-1/H-2/H_2_-3, H-5/H_2_-6/H-7 and H-9/H_2_-10. The HMBC correlations from H_3_-12 to C-3, C-4 and C-5, H_3_-13 to C-7, C-8 and C-9, H_3_-14 to C-10, C-11 and C-15, and H-1 to C-11 and C-15 established the 11-membered ring core structure. The presence of a 4,5-oxirane ring was determined by the chemical shift values of C-4 (*δ*_C_ 64.6) and C-5 (*δ*_C_ 60.7). The NOESY correlations (Figure 3) from Ha-3/H-5, Hb-3/H_3_-12, H_3_-12/H-7, H-7/H-9 and H-9/H_3_-14 indicated the relative configuration as 4R*, 5R*, 7R*, 11R*. The limited quantity did not allow one to define the absolute configuration of **3** through the modified Mosher’s method.

**Figure 5:**
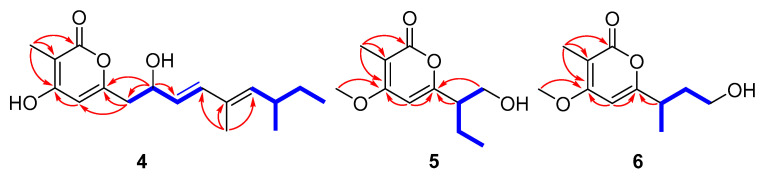
Key HMBC (red arrows) and COSY (blue bold lines) correlations of **4**–**6**.

**Figure 6:**
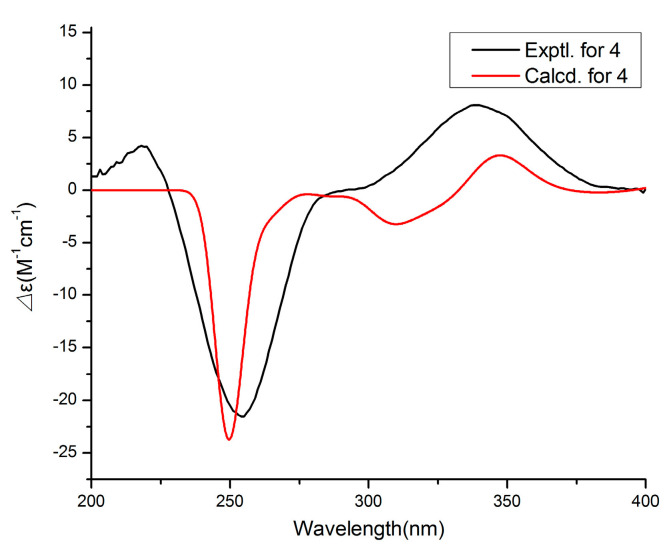
Experimental and calculated ECD spectra of **4**.

Compound **4** was assigned the molecular formula C_17_H_24_O_4_ by the HRESIMS (*m*/*z*: 291.16021 [M − H]^−^). The ^1^H NMR data (Table 2) exhibited the presence of four methyl signals at *δ*_H_ 0.83 (t, *J* = 7.4 Hz, 3H), 0.94 (d *J* = 6.6 Hz, 3H), 1.74 (s, 3H) and 1.85 (s, 3H), and four olefinic proton signals at *δ*_H_ 6.06 (s, 1H), 5.58 (dd, *J* = 6.8, 15.6 Hz, 1H), 6.25 (d, *J* = 15.6 Hz, 1H) and 5.23 (d, *J* = 10.0 Hz, 1H). The ^13^C NMR data revealed 17 carbon resonances including four methyls, two methylenes, six methines (four olefinic carbons) and five other carbons (one carbonyl carbon and two olefinic carbons). Similar NMR data suggested that the structure of **4** was similar to that of proasperfuranone B [15]. The main difference was that the ketone carbonyl group in proasperfuranone B was reduced to a hydroxyl group in **4**. The deduction was confirmed by the HMBC correlations from H-8 to C-6, C-7 and C-9 (Figure 5). Thus, the planar structure of **4** was established. The calculated ECD spectrum fit the experimental spectrum perfectly well (Figure 6) at the BVP86/LANL2MB level in methanol; the absolute configuration of C-8 was determined as 8*R*.

Compound **5**, isolated as a colorless oil, gave a molecular formula of C_11_H_16_O_4_ by HRESIMS data. The ^1^H NMR data (Table 3) exhibited the presence of three methyl signals at *δ*_H_ 0.92 (t, *J* = 7.4 Hz, 3H), 1.91 (s, 3H) and 3.90 (s, 3H), and one olefinic proton at *δ*_H_ 6.10 (s, 1H). The ^13^C NMR data showed 11 carbon resonances assigned to two methyls (*δ*_C_ 8.5, 11.7), one methoxy (*δ*_C_ 56.2), two methylenes (*δ*_C_ 63.6, 22.1), two methines (*δ*_C_ 96.2, 49.4) and four nonprotonated carbons (*δ*_C_ 165.6, 101.3, 163.9 and 165.5). The HMBC correlations from H_3_-11 to C-2, C-3 and C4, and H-5 to C-4 and C-6 revealed the presence of the α-pyrone moiety. The correlations from H-7 and H-8 to C-6, as well as the ^1^H-^1^H COSY cross-peaks of H_2_-8/H-7/H_2_-9/H_3_-10 (Figure 5) indicated the side chain attached to C-6. Thus, the planar structure of **5** was established. By comparing the specific rotation value of **5** (${\left[\alpha \right]}_{D}^{25}$ −32, *c* 0.28, MeOH) with 4-deoxyphomapyrone C (${\left[\alpha \right]}_{D}^{25}$ −40, *c* 0.37, MeOH) [16] and germicidin C (${\left[\alpha \right]}_{D}^{25}$ +21, *c* 0.36, MeOH) [17], the absolute configuration of **5** was assigned as 7*R*.

**Figure 7:**
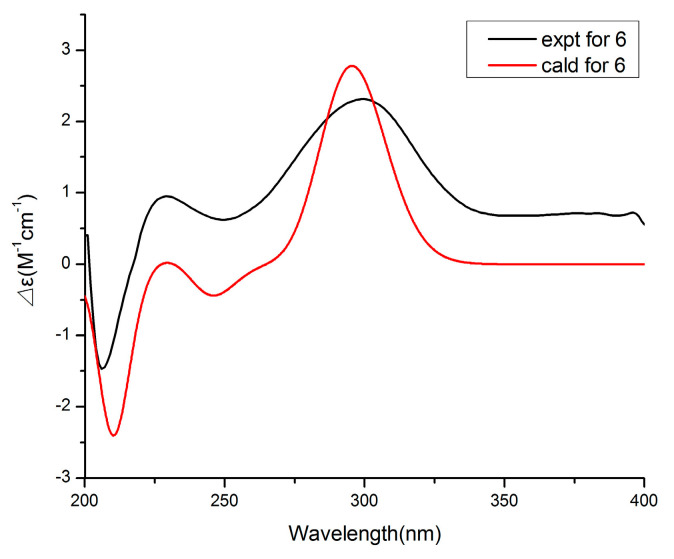
Experimental and calculated ECD spectra of compound **6**.

Compound **6** was obtained as a colorless oil and had a molecular formula of C_11_H_16_O_4_ by HRESIMS. The ^1^H and ^13^C NMR data (Table 3) of **6** were similar to those of **5**, revealing an α-pyrone derivative. Moreover, the planar structure of **6** was established by the spin system of H_3_-10/H-7/H_2_-8/H_2_-9 from ^1^H-^1^H COSY spectra together with the HMBC correlations (Figure 5) from H-7 and H_2_-8 to C-6. Meanwhile, the planar structure of **6** was identified as being the same as phomopyronol [18]. Finally, the calculated ECD spectrum and the experimental data (Figure 7) were well matched, indicating the 7R configuration of **6**.

Compound **7** was identified as 4-*O*-methylgermicidin L (**7**) [12] by a comparison of the spectroscopic data with the literature.

Nitric oxide (NO) is a key biological signaling molecule regulating the variety of physiological functions [19]. The excessive production of NO could induce tissue damage, and it is essential to find new effective NO inhibitors to treat inflammatory diseases and related disorders. Thus, the anti-inflammatory activity of isolated compounds was evaluated against nitric oxide (NO) production in lipopolysaccharide (LPS)-stimulated mouse macrophage RAW 264.7 cells. The results (Table 4 and Table S1) showed that **4** exhibited a potent inhibitory activity with an IC_50_ value of 12.5 μM. Compounds **1**–**2** showed a moderate activity with IC_50_ values of 21.5 and 36.8 μM, respectively, when compared to the positive control (_L_-NMMA, IC_50_: 15.0 μM). All the tested compounds were nontoxic at the tested concentration.

## 3. Experimental Section

### 3.1. General Experimental Procedures

Specific rotations were taken on a MCP 300 (Anton Paar) polarimeter at 28 °C. UV spectra were recorded in MeOH using a PERSEE TU-1900 spectrophotometer, and ECD data were measured on a Chirascan CD spectrometer (Applied Photophysics). IR spectra were obtained on a Nicolet Nexus 670 spectrophotometer, in KBr discs. All NMR experiments were performed on a Bruker Avance 500 spectrometer at room temperature. HRESIMS spectra were obtained on a Thermo Fisher Scientific Q-TOF mass spectrometer. Column chromatography (CC) was conducted using silica gel (200–300 mesh, Qingdao Marine Chemical Factory) and Sephadex LH-20 (Amersham Pharmacia). Semipreparative HPLC was carried out using a C18 column (ODS, 250 × 10 mm, 5 μm). Thin-layer chromatography (TLC) was performed on silica gel plates (Qingdao Huang Hai Chemical Group Co., G60, F-254).

### 3.2. Fungal Material, Fermentation and Isolation

The strain QYM12 was isolated from the healthy leaves of *Kandelia candel*, which were collected in June 2017 from the South China Sea, Dongzhai Harbor Mangrove Nature Reserve Area, Hainan Province, China. Fungal identification was achieved using a molecular biological protocol by DNA amplification and ITS sequence [20]. The sequence was the most similar (99%) to the sequence of *Diaporthe* sp. (GU066666.1) via BLAST research. The sequence data of the strain has been deposited at GenBank with the accession number MW332459. The fungus was preserved at Sun Yat-Sen University, China. The strain was cultured on PDA medium for four days. Then, the seed culture was prepared by the mycelia of the fungus being inoculated into 500 mL of PDB medium for five days. Thereafter, the seed culture was transferred into solid rice medium (800 × 1000 Erlenmeyer flasks each containing 80 g of raw rice and 70 mL of 0.3% seawater) at 28 °C for 30 days.

Thereafter, the fermented material was extracted with MeOH three times, and organic phases were combined and evaporated under reduced pressure to yield an extract of 25.0 g. Then, the residue was fractionated by silica gel column chromatography with a gradient of petroleum ether/EtOAc from 10:0 to 0:10 to give eight fractions (Fr.1‒Fr.8, per 10 mL). Fr.3 (380.0 mg) was subjected to Sephadex LH-20 CC (CH_2_Cl_2_/MeOH *v*/*v*, 1:1) to yield three fractions (3.1–3.3). Fr.3.1 (10.0 mg) was purified by silica gel CC (CH_2_Cl_2_/MeOH *v*/*v*, 75:1) to yield compound **1** (3.5 mg). Fr.4 (565.0 mg) was subjected to Sephadex LH-20 CC (CH_2_Cl_2_/MeOH *v*/*v*, 1:1) to yield two fractions (4.1 and 4.2). Fr.4.1 (36.5 mg) was purified by semipreparative reversed-phase HPLC (MeOH‒H_2_O, 50:1) to yield compound **7** (3.1 mg). Fr.4.2 (46.2 mg) was subjected to silica gel CC (CH_2_Cl_2_/MeOH *v*/*v*, 95:5) to yield compounds **2** (2.0 mg) and **5** (5.6 mg). Fr.5 (522.0 mg) was purified by Sephadex LH-20 CC (CH_2_Cl_2_/MeOH *v*/*v*, 1:1) to afford three fractions (5.1–5.3). Fr.5.1 (7.6 mg) was subjected to silica gel CC (CH_2_Cl_2_/MeOH *v*/*v*, 25:1) to give compound **3** (2.0 mg). Fr.6 (650.0 mg) was subjected to Sephadex LH-20 CC (CH_2_Cl_2_/MeOH *v*/*v*, 1:1) to give four fractions (6.1–6.4). Fr.6.1 (38.0 mg) was purified by silica gel CC (CH_2_Cl_2_/MeOH *v*/*v*, 10:1) to yield compound **6** (6.8 mg). Fr.6.2 (15.0 mg) was subjected to silica gel CC (CH_2_Cl_2_/MeOH *v*/*v*, 17:3) to yield compound **4** (3.3 mg).

Diaporpenoid A (**1**): colorless oil; ${\left[\alpha \right]}_{D}^{25}$ −32 (*с* 0.46, MeOH); UV (MeOH) *λ*_max_ (log *ε*): 215 (2.52) nm; IR (KBr) *ν*_max_: 3376, 2910, 2896, 1685, 1413, 1352, 1206, 1026 cm^−1^; ^1^H and ^13^C NMR (500 MHz, CDCl_3_) data, Table 1; HRESIMS *m*/*z* 391.20900 [M + Na]^+^ (calcd for C_20_H_32_O_6_Na, 391.20911).

Diaporpenoid B (**2**): colorless oil; ${\left[\alpha \right]}_{D}^{25}$ +28 (*с* 0.06, CDCl_3_); UV (MeOH) *λ*_max_ (log *ε*): 209 (1.86), 281 (3.02) nm; IR (KBr) *ν*_max_: 3422, 3268, 2798, 1632, 1590, 1330, 1215, 1063 cm^−1^; ^1^H and ^13^C NMR (500 MHz, CDCl_3_) data, see Table 1; HRESIMS *m*/*z* 357.24244 [M + H]^+^ (calcd for C_20_H_32_O_6_, 357.24242).

Diaporpenoid C (**3**): colorless oil; ${\left[\alpha \right]}_{D}^{25}$ +18 (*с* 0.04, MeOH); UV (MeOH) *λ*_max_ (log *ε*): 220 (2.52) nm; IR (KBr) *ν*_max_: 3320, 1762, 1525, 1376, 1356, 1132, 1010 cm^−1^; ^1^H and ^13^C NMR (500 MHz, MeOH-*d*_4_) data, see Table 2; HRESIMS *m*/*z* 265.14401 [M − H]^−^ (calcd for C_15_H_22_O_4_, 265.14453).

Diaporpyrane A (**4**): colorless oil; ${\left[\alpha \right]}_{D}^{25}$ +12 (*с* 0.07, MeOH); UV (MeOH) *λ*_max_ (log *ε*): 212 (3.22), 240 (3.53) nm; IR (KBr) *ν*_max_: 3420, 2986, 2855, 1762, 1727, 1612, 1344, 1235, 1086 cm^−1^; ^1^H and ^13^C NMR (500 MHz, MeOH-*d*_4_) data, see Table 2; HRESIMS *m*/*z* 291.16021 [M − H]^−^ (calcd for C_17_H_24_O_4_, 291.16018).

Diaporpyrane B (**5**): colorless oil; ${\left[\alpha \right]}_{D}^{25}$ −32, (*с* 0.28, MeOH); UV (MeOH) *λ*_max_ (log *ε*): 212 (3.45), 283 (3.62) nm; IR (KBr) *ν*_max_: 3176, 2965, 1647, 1580, 1421 cm^−1^; ^1^H and ^13^C NMR (500 MHz, CDCl_3_) data, see Table 3; HRESIMS *m*/*z* 213.11221 [M + H]^+^ (calcd for 213.11214, C_11_H_17_O_4_).

Diaporpyrane C (**6**): colorless oil; ${\left[\alpha \right]}_{D}^{25}$ −65 (*с* 0.85, MeOH); UV (MeOH) *λ*_max_ (log *ε*): 205(3.32), 300 (3.67) nm; IR (KBr) *ν*_max_: 3445, 2962, 1735, 1675, 1363, 1256, 1218 cm^−1^; ^1^H and ^13^C NMR (500 MHz, CDCl_3_) data, see Table 3; HRESIMS *m*/*z* 213.1116 [M + H]^+^ (calcd for C_11_H_17_O_4_, 213.1117).

### 3.3. ECD Calculation Methods

The calculation was accomplished according to the method described previously [21]. The conformers of compounds **1**, **4** and **6** were first optimized by DFT methods at the B3LYP/6-31G (d) level in the Gaussian 05 program. Then, the theoretical calculation was performed using the time-dependent density functional theory (TD-DFT) at the level of B3LYP/DGDZVP, BVP86/LANL2MB and B3LYP/DGTZVP, respectively.

### 3.4. Anti-Inflammatory Assay

The RAW264.7 cells were purchased from Macrophage Resource Center, Shanghai Institute of Life Sciences, Chinese Academy of Sciences (Shanghai, China). The method for the assay of the anti-inflammatory activity was conducted according to a previously published paper [20]. The detailed process is described in the Supplementary Materials. 

## 4. Conclusions

In summary, the strain *Diaporthe* sp. QYM12, which was isolated from *Kandelia candel*, Dongzhai Harbor Mangrove Nature Reserve Area, was cultured in solid rice medium, leading to the identification of six new metabolite diaporpenoids A‒C (**1**–**3**) and diaporpyrones A‒C (**4**–**6**). Compound **1** was a macrocyclic diterpenoid featuring a rare 5/10/5-fused tricyclic ring system, and compounds **2**,**3** were macrocyclic sesquiterpenoids possessing a hendecane core. Macrocyclic sesquiterpenoids and diterpenoids are a functionally diverse group of natural products with versatile bioactivities [22]. For instance, junceellolide C showed an anti-HBV activity [23], flaccidenol A displayed a cytotoxic activity [24], antipacid B exhibited an anti-inflammatory activity [25], and euphorbesulins A revealed an antimalarial activity [26]. The anti-inflammatory assay suggested that compound **1** showed a moderate activity with an IC_50_ value of 21.5 μM. Compound **4** exhibited a potent inhibitory activity with an IC_50_ value of 12.5 μM. Proinflammatory enzymes, including nitric oxide synthase (iNOS) and cyclooxygenase-2 (COX-2), were reported to play key roles in inflammatory processes [27]. Thus, further research is required to clarify the underlying mechanisms of the active compounds. This study has suggested that these macrocyclic sesquiterpenoids and *α*-pyrone derivatives have the potential to develop lead compounds for anti-inflammatory agents.

## Figures and Tables

**Table 1:** ^1^H and ^13^C NMR data for Compounds **1** and **2** in CDCl_3_.

No.	1		2	
	*δ*_C_, Type	*δ*_H_ (*J* in Hz)	*δ*_C_, Type	*δ*_H_ (*J* in Hz)
1	80.9, C		137.8, C	
2	46.4, CH_2_	2.16, m	108.9, CH	6.26, d (2.2)
3	81.5, CH	4.95, td (2.5, 7.3)	154.4, C	
4	54.1, CH	2.16, m	101.4, CH	6.18, d (2.3)
4a			154.5, C	
5	50.6, CH	1.96, dt (6.8, 13.2)	79.4, C	
6*α*	81.2, C		42.8, CH_2_	2.51, d (14.5)
6*β*				2.22, m
7	46.0, CH_2_	2.21, m	121.3, CH	5.14, m
		2.05, m		
8	81.0, CH	4.86, td (2.5, 6.8)	141.4, CH	5.15, d (2.2)
9	49.6, CH	2.56, dt (7.2, 10.0)	38.5, C	
10	44.0, CH	2.05, m	40.6, CH_2_	2.23, m
				1.77, m
11	23.7, CH_3_	1.20, s	123.5, CH	5.17, m
12	181.1, C		138.6, C	
13	42.6, CH	2.72, qd (3.1, 7.6)	78.2, CH	3.99, d (9.6)
14*α*	18.3, CH_3_	1.33, d (7.6)	39.7, CH_2_	1.76, m
14*β*				1.11, dd (9.3, 13.5)
14a			34.2, CH	1.69, m
15*α*	15.9, CH_3_	0.97, d (7.3)	27.3, CH_2_	2.88, dd (5.6, 16.4)
15*β*				2.24, m
15a			112.7, C	
16	23.8, CH_3_	1.22, s	19.3, CH_3_	2.19, s
17	180.5, C		19.8, CH_3_	1.06, s
18	38.3, CH	2.90, dq (7.3, 9.9)	24.1, CH_3_	1.01, s
19	11.6, CH_3_	1.31, d (7.3)	30.4, CH_3_	1.06, s
20	15.8, CH_3_	0.99, d (7.2)	10.6, CH_3_	1.65, s

**Table 2:** ^1^H and ^13^C NMR data for **3** and **4** in MeOH-*d*_4_.

No.	3			4	
	*δ*_C_, Type	*δ*_H_ (*J* in Hz)	No.	*δ*_C_, Type	*δ*_H_ (*J* in Hz)
1	138.8, CH	5.50, d (15.8)	2	167.7, C	
2	124.6, CH	5.45, ddd (4.7, 10.6, 15.8)	3	97.8, C	
3*α*	44.1, CH_2_	2.60, dd (4.7, 11.9)	4	166.4, C	
3*β*		1.57, dd (10.6, 11.9)	5	101.7, CH	6.06, s
4	64.6, C		6	160.0, C	
5	60.7, CH	2.45, dd (5.2, 9.7)	7	41.4, CH_2_	2.65, m
6*α*	34.4, CH_2_	2.19, ddd (5.1, 10.0, 13.3)	8	69.8, CH	4.47, d (6.5)
6*β*		1.61, m	9	127.7, CH	5.58, dd (6.8, 15.6)
7	76.4, CH	4.10, dd (6.6, 10.1)	10	135.8, CH	6.25, d (15.6)
8	137.3, C		11	131.6, C	
9	126.5, CH	5.16, brd (11.4)	12	139.6, CH	5.23, d (10.0)
10*α*	36.4, CH_2_	2.71, dd (12.2, 13.3)	13	34.2, CH	2.40, m
10*β*		2.08, brd (12.2)	14*α*	30.1, CH_2_	1.38, m
11	49.1, C		14*β*		1.24, m
12	17.0, CH_3_	1.34, s	15	10.9, CH_3_	0.83, t (7.4)
13	10.8, CH_3_	1.64, s	16	19.6, CH_3_	0.94, d (6.6)
14	19.7, CH_3_	1.39, s	17	11.5, CH_3_	1.74, s
15	181.5, C		18	6.8, CH_3_	1.85, s

**Table 3:** ^1^H and ^13^C NMR data for **5**–**7** in CDCl_3_.

No.	5		6	
	*δ*_C_, Type	*δ*_H_ (*J* in Hz)	*δ*_C_, Type	*δ*_H_ (*J* in Hz)
2	165.6, C		166.2, C	
3	101.3, C		101.2, C	
4	163.9, C		166.2, C	
5	96.2, CH	6.10, s	93.8, CH	6.25, s
6	165.5, C		167.6, C	
7	49.4, CH	2.56, m	35.5, CH	2.82, dq (6.8, 13.7)
8	63.6, CH_2_	3.88, m	37.4, CH_2_	1.93, m
				1.75, dt (6.1, 13.6)
9	22.1, CH_2_	1.65, m	60.3, CH_2_	3.63, m
10	11.7, CH_3_	0.92, t (7.4)	18.7, CH_3_	1.25, d (6.9)
11	8.5, CH_3_	1.91, s	8.5, CH_3_	1.87, s
12	56.2, CH_3_	3.90, s	56.4, CH_3_	3.86, s

**Table 4:** The anti-inflammatory activities of compounds **1**–**8**.

Compound	1	2	3	4	5	6	7	_L_-NMMA ^a^
IC_50_ (μM)	21.5	36.8	50.0	12.5	-	-	50.0	15.0

- not tested. ^a^ positive control.

## Data Availability

Data is contained within the article and Supplementary Material.

## Supplementary Materials

The following are available online at https://www.mdpi.com/1660-3397/19/2/56/s1. Figure S1: ^1^H NMR spectrum of compound **1** (500 MHz, CDCl_3_). Figure S2: ^13^C NMR spectrum of compound **1** (125 MHz, CDCl_3_). Figure S3: HSQC spectrum of compound **1**. Figure S4: ^1^H-^1^H COSY spectrum of compound **1**. Figure S5: HMBC spectrum of compound **1**. Figure S6: HRESIMS spectrum of compound **1**. Figure S7: NOESY spectrum of compound **1**. Figure S8: ^1^H NMR spectrum of compound **2** (500 MHz, CDCl_3_). Figure S9: ^13^C NMR spectrum of compound **2** (125 MHz, CDCl_3_). Figure S10: HSQC spectrum of compound **2**. Figure S11: ^1^H-^1^H COSY spectrum of compound **2**. Figure S12: HMBC spectrum of compound **2**. Figure S13: NOESY spectrum of compound **2**. Figure S14: HRESIMS spectrum of compound **2**. Figure S15: ^1^H NMR spectrum of compound **3** (500 MHz, MeOH-*d*_4_). Figure S16: ^13^C NMR spectrum of compound **3** (125 MHz, MeOH-*d*_4_). Figure S17: HSQC spectrum of compound **3**. Figure S18: ^1^H-^1^H COSY spectrum of compound 3. Figure S19: HMBC spectrum of compound 3. Figure S20: NOESY spectrum of compound **3**. Figure S21: HRESIMS spectrum of compound **3**. Figure S22: ^1^H NMR spectrum of compound **4** (500 MHz, MeOH-*d*_4_). Figure S23: ^13^C NMR spectrum of compound **4** (125 MHz, MeOH-*d*_4_). Figure S24: HSQC spectrum of compound **4**. Figure S25: ^1^H-^1^H COSY spectrum of compound **4**. Figure S26: HMBC spectrum of compound **4**. Figure S27: HRESIMS spectrum of compound **4**. Figure S28: ^1^H NMR spectrum of compound **5** (500 MHz, CDCl_3_). Figure S29: ^13^C NMR spectrum of compound **5** (125 MHz, CDCl_3_). Figure S30: HSQC spectrum of compound **5**. Figure S31: ^1^H-^1^H COSY spectrum of compound **5**. Figure S32: HMBC spectrum of compound **5**. Figure S33: HRESIMS spectrum of compound **5**. Figure S34: ^1^H NMR spectrum of compound **6** (500 MHz, CDCl_3_). Figure S35: ^13^C NMR spectrum of compound **6** (125 MHz, CDCl_3_). Figure S36: HSQC spectrum of compound **6**. Figure S37: ^1^H-^1^H COSY spectrum of compound **6**. Figure S38: HMBC spectrum of compound **6**. Figure S39: HRESIMS spectrum of compound **6**.
