# Supplementary material for: Metabolites with Anti-Inflammatory Activity from the Mangrove Endophytic Fungus Diaporthe sp. QYM12
Source: Mar Drugs. 2021 Jan 24;19(2):56. doi: 10.3390/md19020056 (PMC7912375; doi:10.3390/md19020056)
Supplement: Supplementary file 1 [file marinedrugs-19-00056-s001.pdf]

Metabolites with Anti-inflammatory Activity from the Mangrove Endophytic Fungus *Diaporthe* sp. QYM12

Yan Chen [1,2], Ge Zou [2], Wencong Yang [2], Yingying Zhao [1], Qi Tan [2], Lin Chen [3], Jinmei Wang [1], Changyang Ma [1], Wenyi Kang [1,*] and Zhigang She [2,*]

[1] National R & D Center for Edible Fungus Processing Technology, Henan University, Kaifeng 475004, China; chenyan27@mail2.sysu.edu.cn (Y.C.); zhaoyingying@vip.henu.edu.cn (Y.Z.); wangjinmei@henu.edu.cn (J.W.); macaya1024@vip.henu.edu.cn (C.M.);

[2] School of Chemistry, Sun Yat-Sen University, Guangzhou 510275, China; zoug5@mail2.sysu.edu.cn (G.Z.); yangwc6@mail2.sysu.edu.cn (W.Y.); tanq27@mail2.sysu.edu.cn (Q.T.);

[3] Henan Joint International Research Laboratory of Drug Discovery of Small Molecules, Zhengzhou Key Laboratory of Synthetic Biology of Natural Products, Huanghe Science and Technology College, Zhengzhou 450063, China; lchenchina@hhstu.edu.cn (L.C.)

* Correspondence: kangwenyi@henu.edu.cn (W.K.); cesshzhg@mail.sysu.edu.cn (Z.S.)

Supporting Information Contents:

Figure. S1 ^{1}H NMR spectrum of compound **1** (500 MHz, $CDCl_3$).

Figure. S2 ^{13}C NMR spectrum of compound **1** (125 MHz, $CDCl_3$).

Figure. S3 HSQC spectrum of compound **1**.

Figure. S4 ^{1}H-^{1}H COSY spectrum of compound **1**.

Figure. S5 HMBC spectrum of compound **1**.

Figure. S6 HRESIMS spectrum of compound **1**.

Figure. S7 NOESY spectrum of compound **1**.

Figure. S8 ^{1}H NMR spectrum of compound **2** (500 MHz, $CDCl_3$).

Figure. S9 ^{13}C NMR spectrum of compound **2** (125 MHz, $CDCl_3$).

Figure. S10 HSQC spectrum of compound **2**.

Figure. S11 ^{1}H-^{1}H COSY spectrum of compound **2**.

Figure. S12 HMBC spectrum of compound **2**.

Figure. S13 NOESY spectrum of compound **2**.

Figure. S14 HRESIMS spectrum of compound **2**.

Figure. S15 ^{1}H NMR spectrum of compound **3** (500 MHz, MeOH-d_4).

Figure. S16 ^{13}C NMR spectrum of compound **3** (125 MHz, MeOH-d_4).

Figure. S17 HSQC spectrum of compound **3**.

Figure. S18 ^{1}H-^{1}H COSY spectrum of compound **3**.

Figure. S19 HMBC spectrum of compound **3**.

Figure. S20 NOESY spectrum of compound **3**.

Figure. S21 HRESIMS spectrum of compound **3**.

Figure. S22 ^{1}H NMR spectrum of compound **4** (500 MHz, MeOH-d_4).

Figure. S23 ^{13}C NMR spectrum of compound **4** (125 MHz, MeOH-d_4).

Figure. S24 HSQC spectrum of compound **4**.

Figure. S25 ^{1}H-^{1}H COSY spectrum of compound **4**.

Figure. S26 HMBC spectrum of compound **4**.

Figure. S27 HRESIMS spectrum of compound **4**.

Figure. S28 ^{1}H NMR spectrum of compound **5** (500 MHz, $CDCl_3$).

Figure. S29 ^{13}C NMR spectrum of compound **5** (125 MHz, $CDCl_3$).

Figure. S30 HSQC spectrum of compound **5**.

Figure. S31 ^{1}H-^{1}H COSY spectrum of compound **5**.

Figure. S32 HMBC spectrum of compound **5**.

Figure. S33 HRESIMS spectrum of compound **5**.

Figure. S34 ^{1}H NMR spectrum of compound **6** (500 MHz, $CDCl_3$).

Figure. S35 ^{13}C NMR spectrum of compound **6** (125 MHz, $CDCl_3$).

Figure. S36 HSQC spectrum of compound **6**.

Figure. S37 ^{1}H-^{1}H COSY spectrum of compound **6**.

Figure. S38 HMBC spectrum of compound **6**.

Figure. S39 HRESIMS spectrum of compound **6**.

1. The method of anti-inflammatory assay.

Table S1. Inhibitory activities against LPS-Induced NO Production of **1-7** at 50 μM.

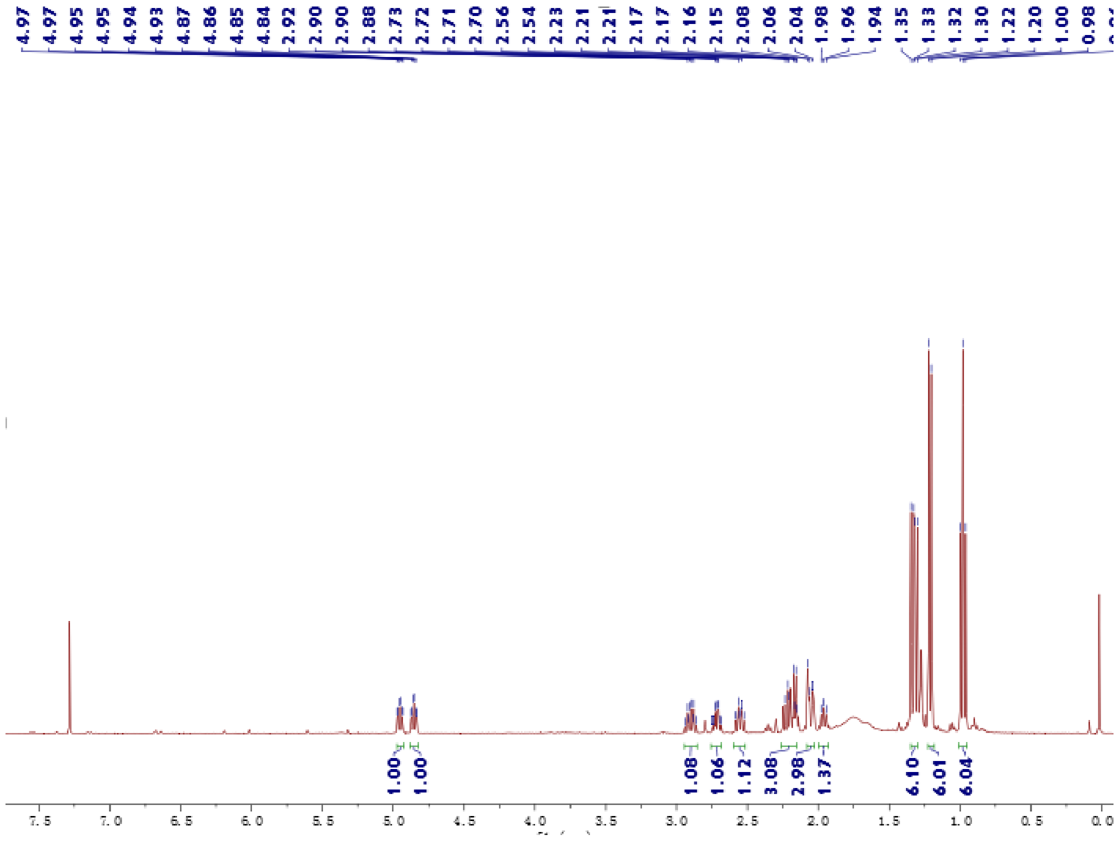

Figure. S1 ^{1}H NMR spectrum of compound **1** (500 MHz, $CDCl_3$).

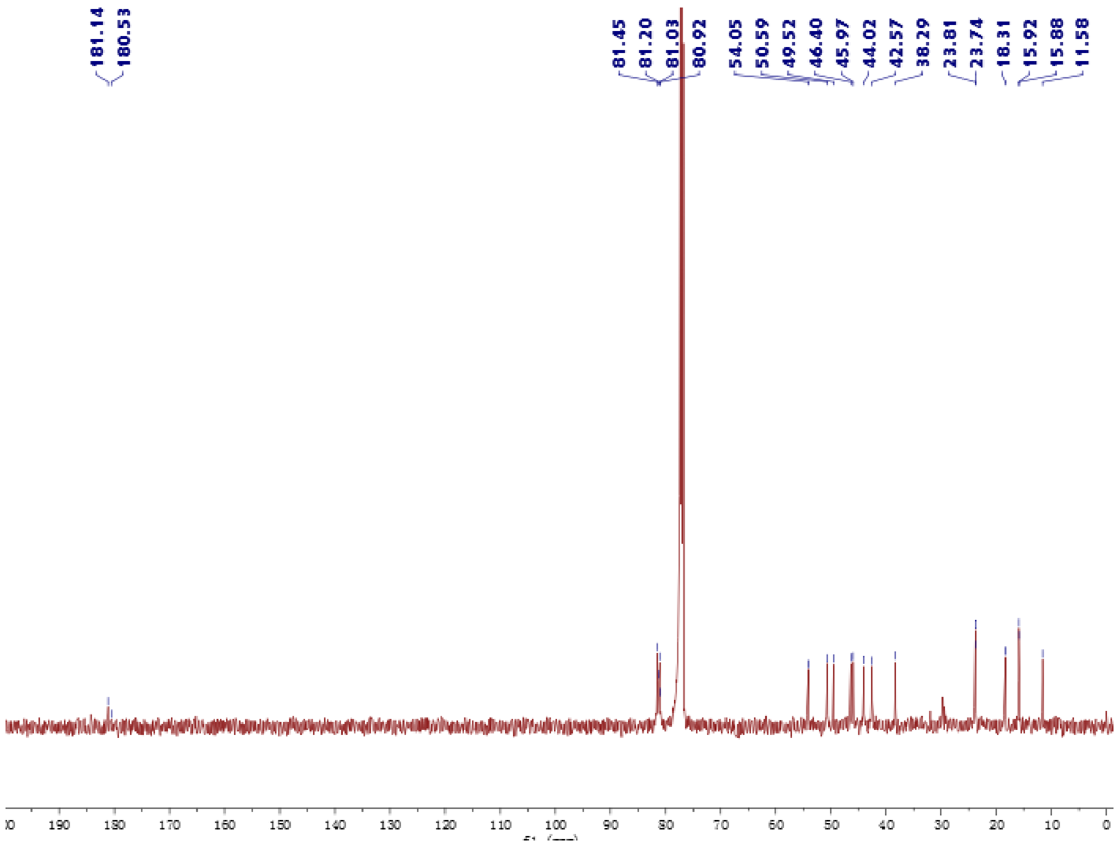

Figure. S2 ^{13}C NMR spectrum of compound **1** (125 MHz, $CDCl_3$).

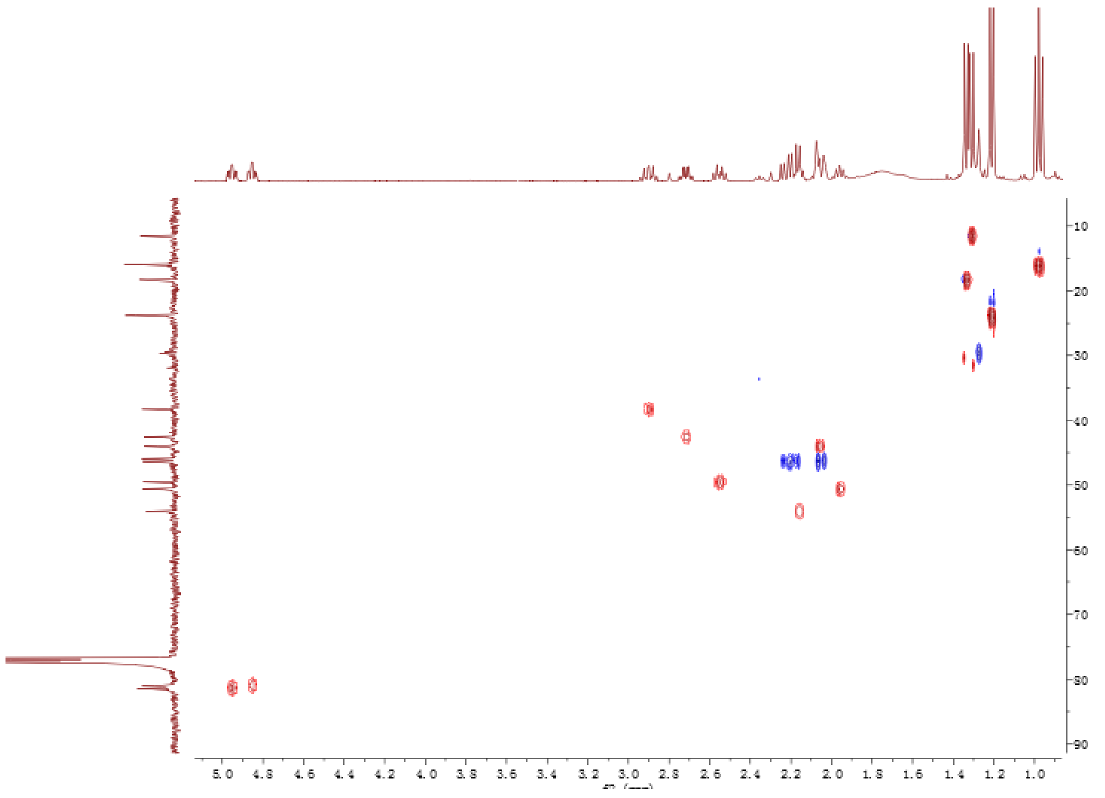

Figure. S3 HSQC spectrum of compound **1**.

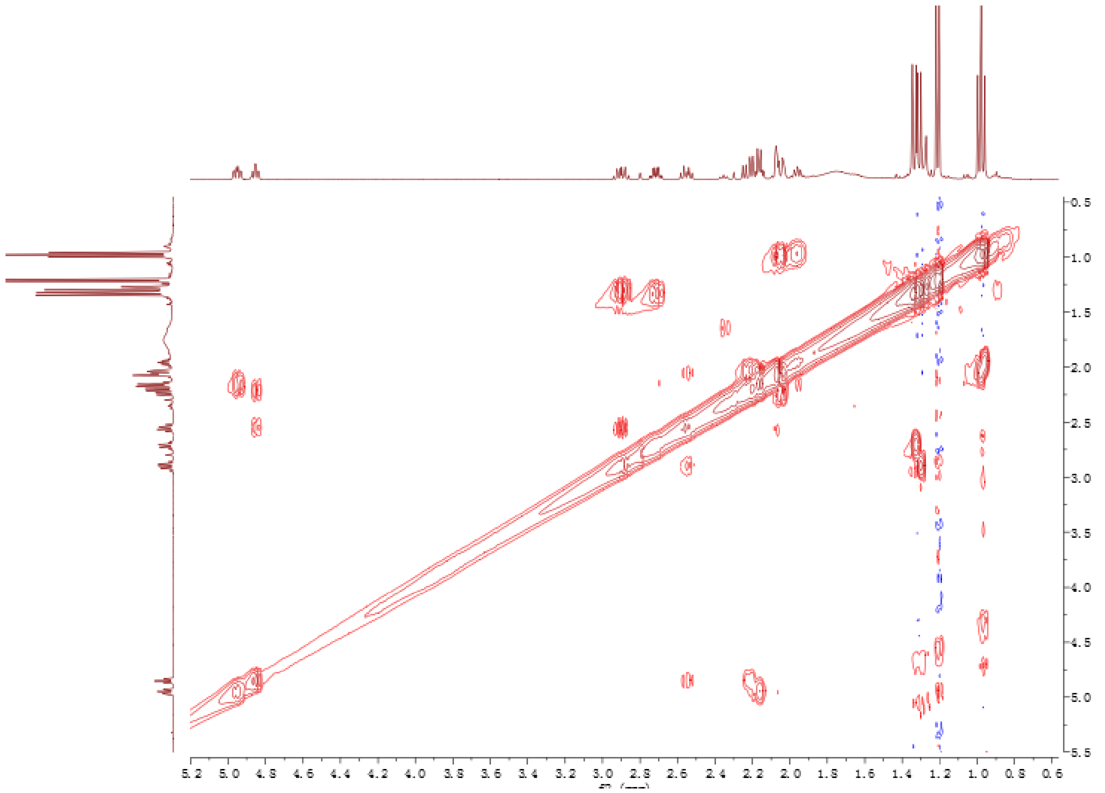

Figure. S4 ^{1}H-^{1}H COSY spectrum of compound **1**.

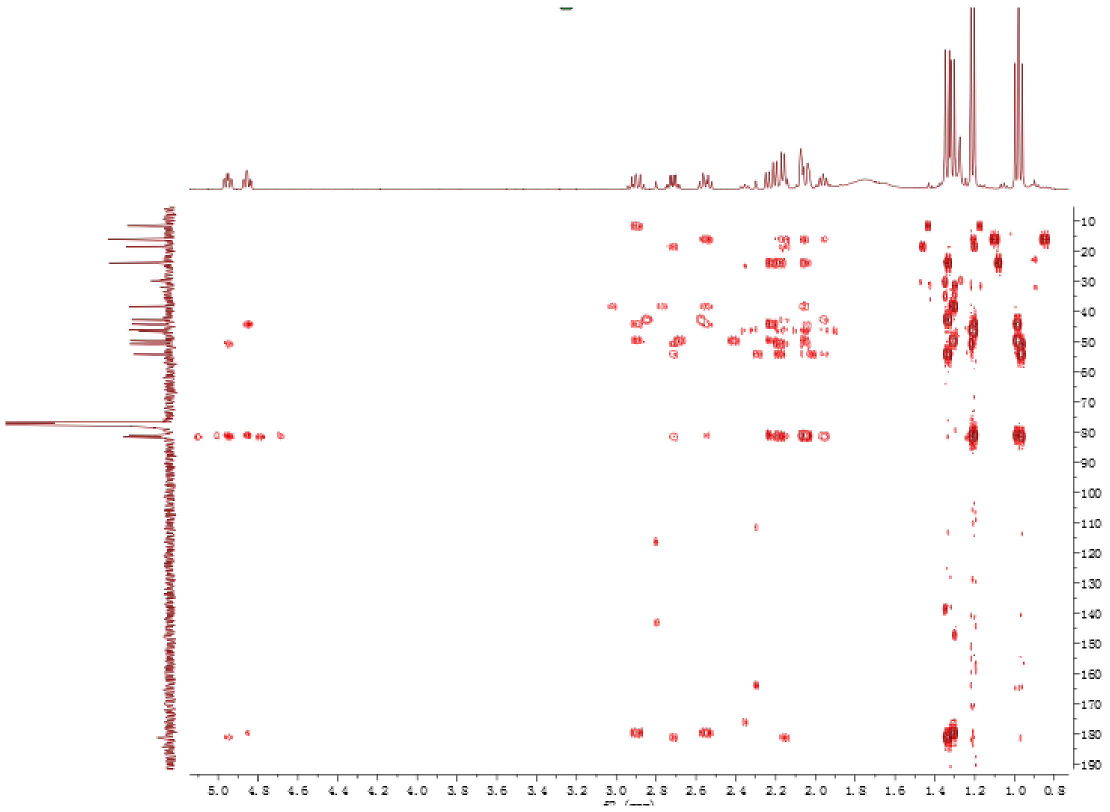

Figure. S5 HMBC spectrum of compound **1**.

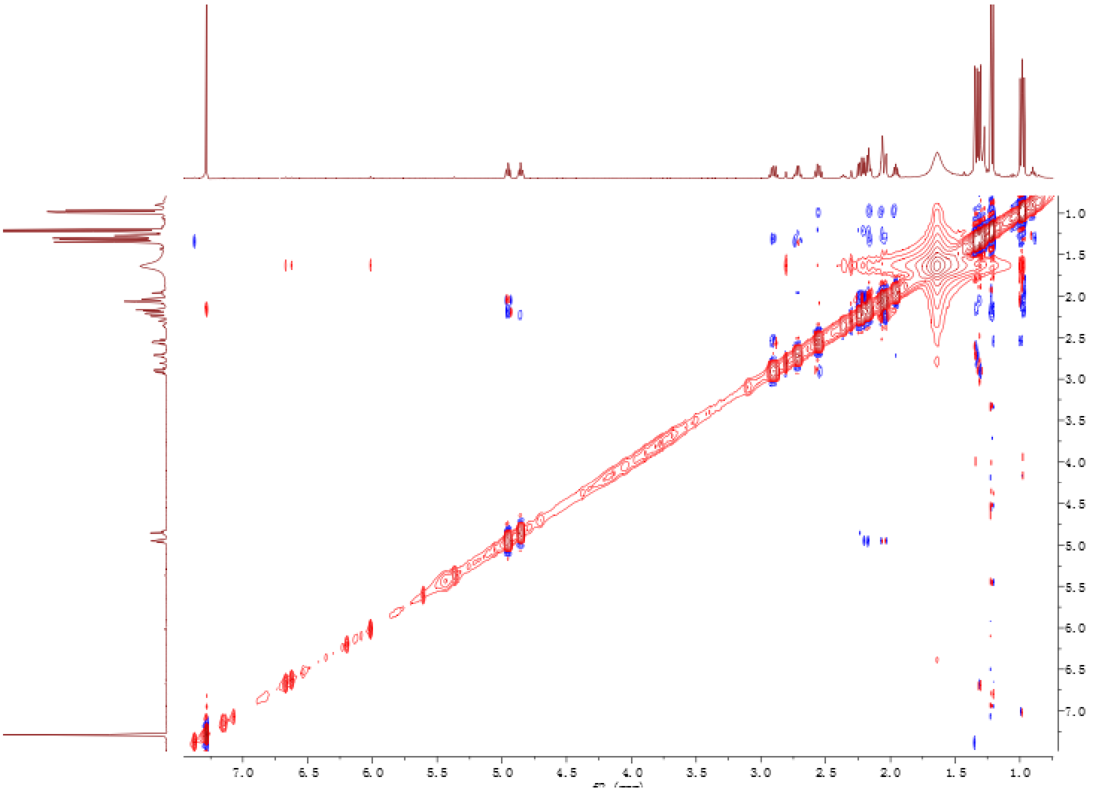

Figure. S6 NOESY spectrum of compound **1**.

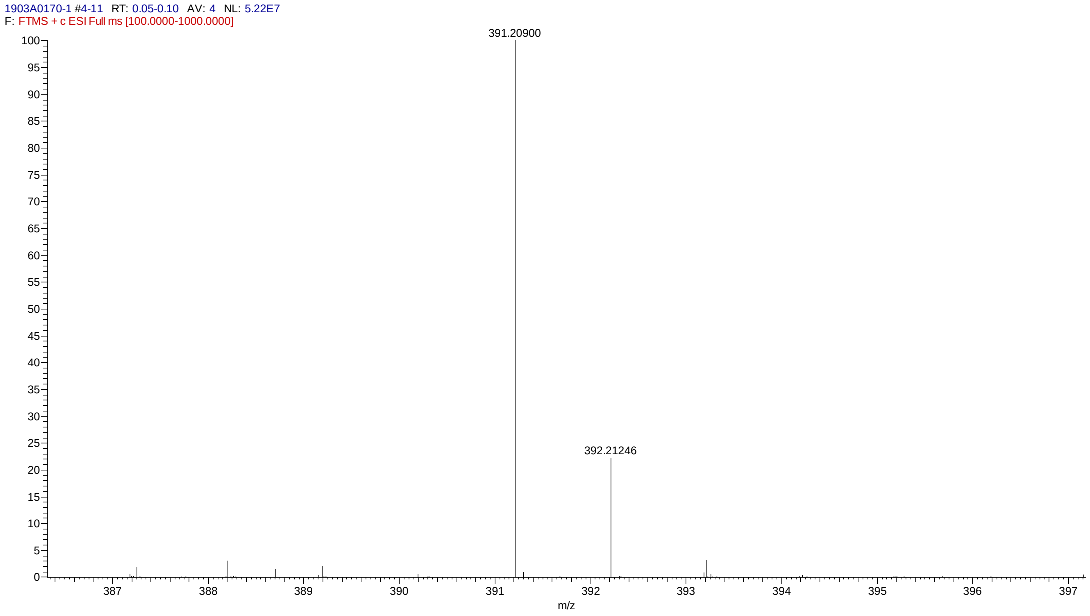

Figure. S7 HRESIMS spectrum of compound **1**.

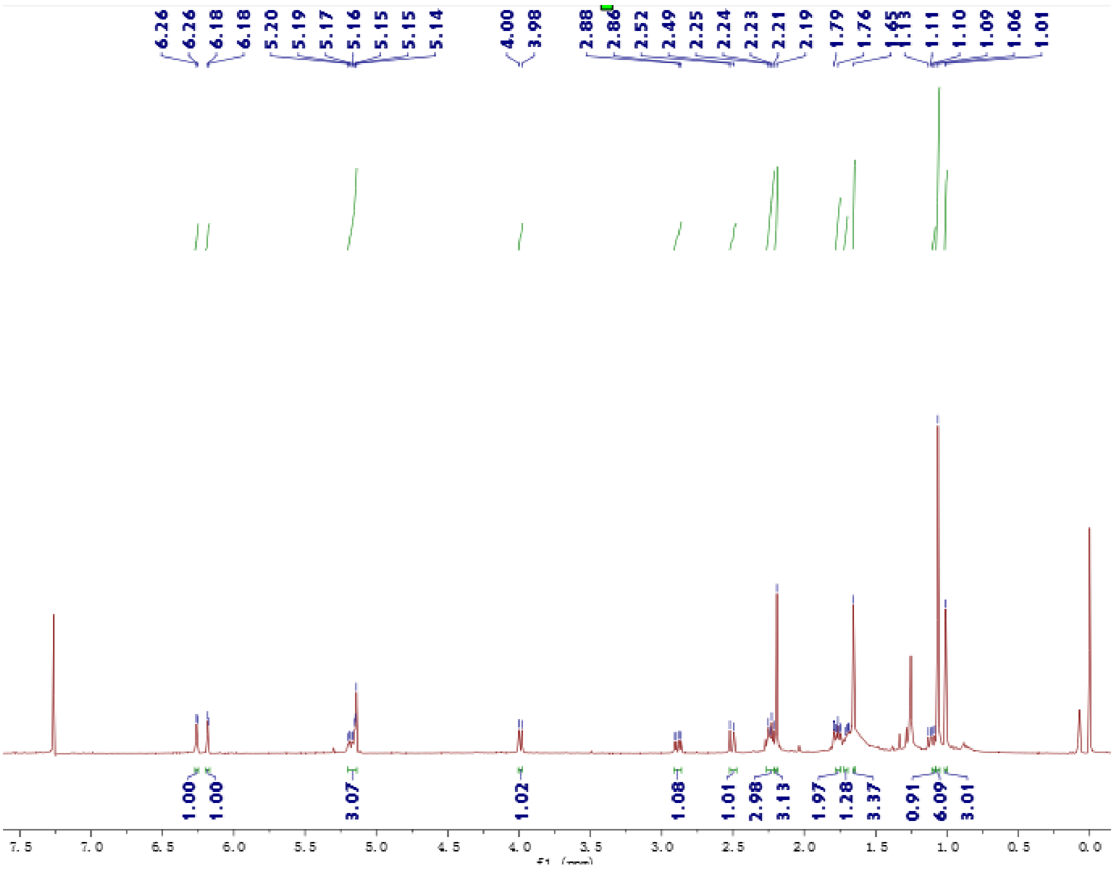

Figure. S8 ^{1}H NMR spectrum of compound **2** (500 MHz, $CDCl_3$).

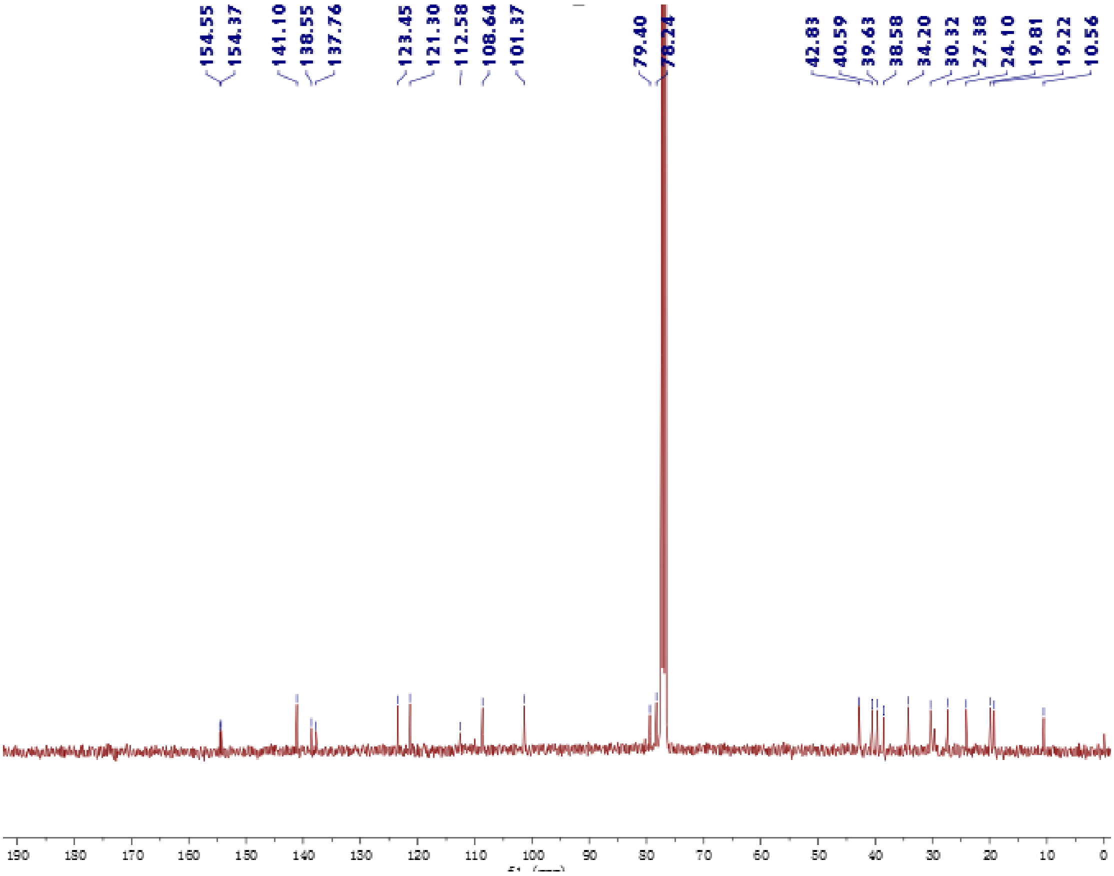

Figure. S9 ^{13}C NMR spectrum of compound **2** (125 MHz, $CDCl_3$).

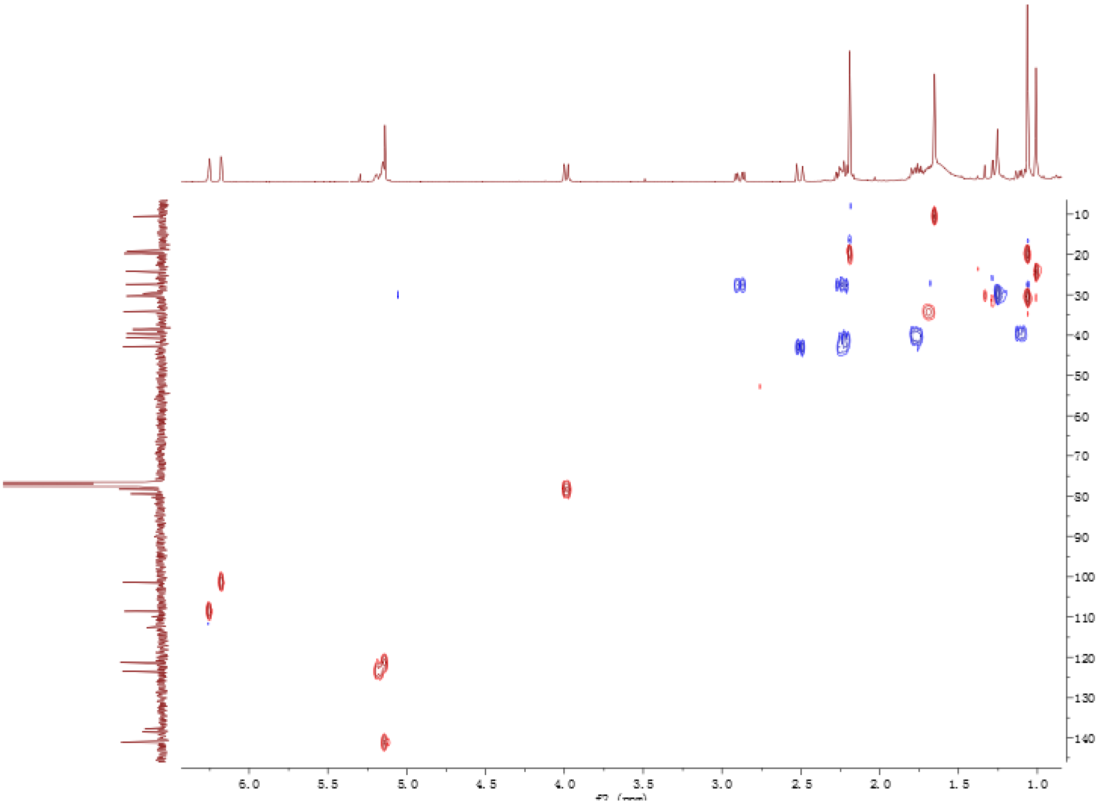

Figure. S10 HSQC spectrum of compound **2**.

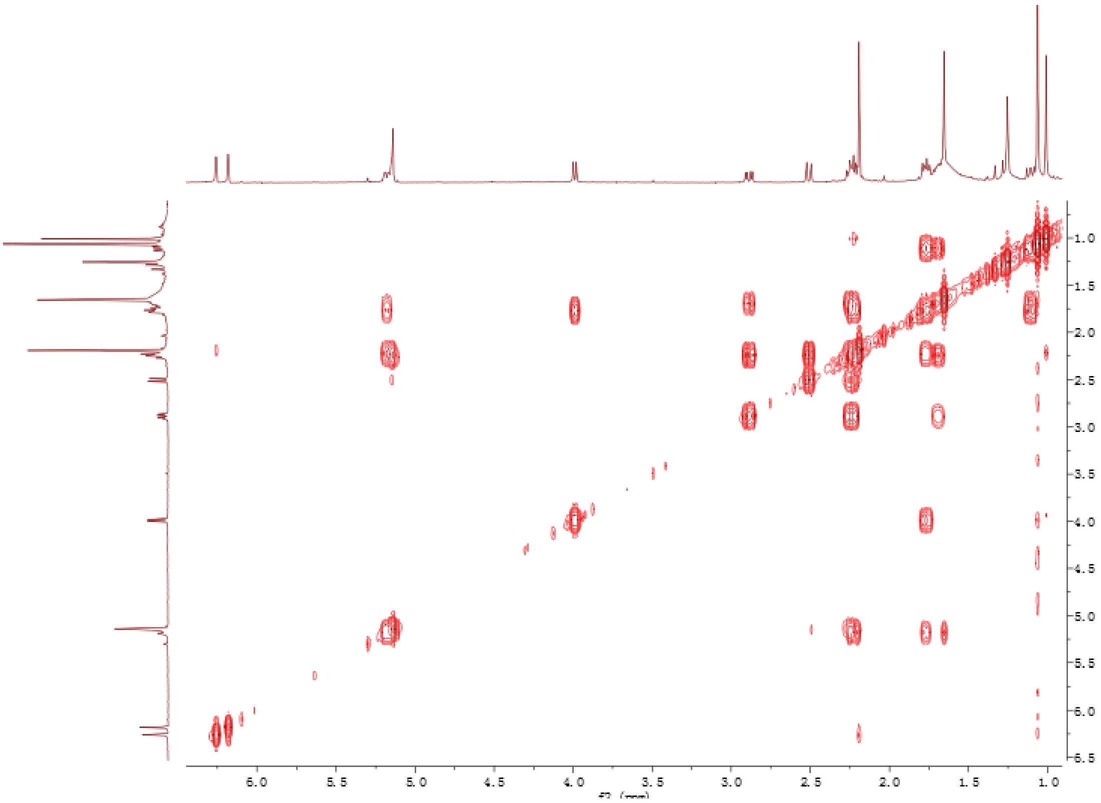

Figure. S11 ${}^{1}H$-${}^{1}H$ COSY spectrum of compound **2**.

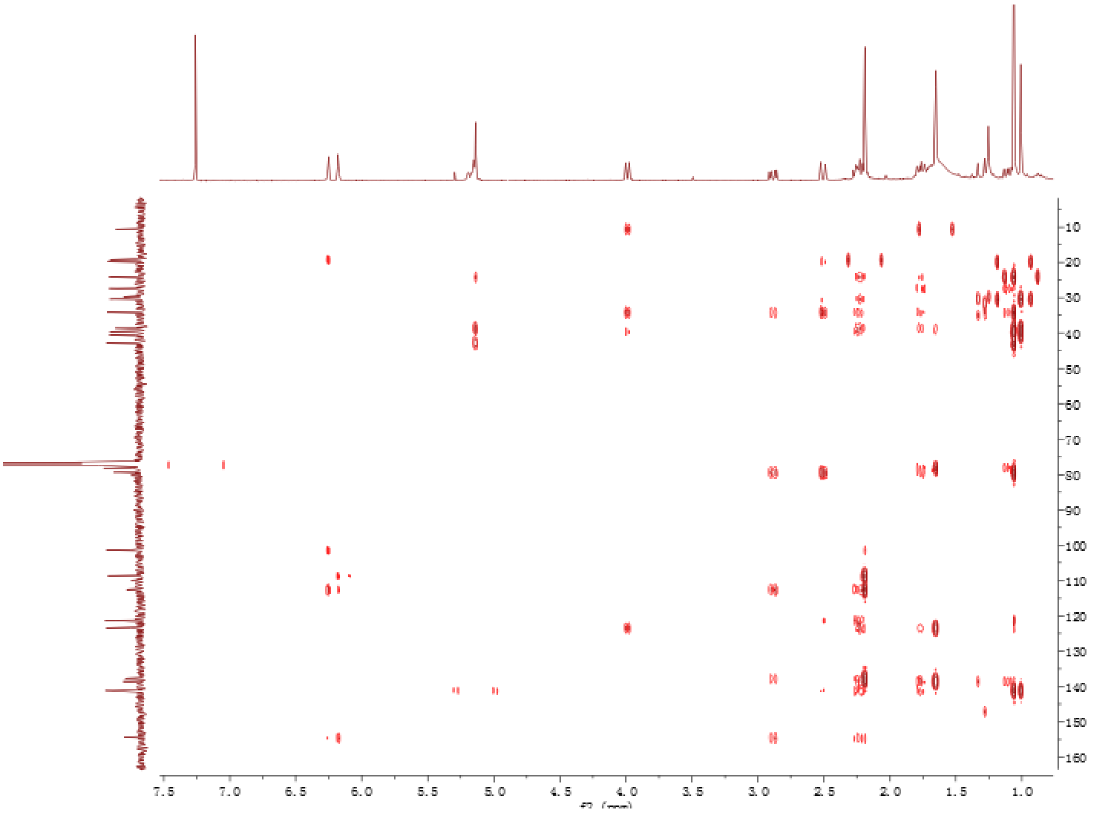

Figure. S12 HMBC spectrum of compound **2**.

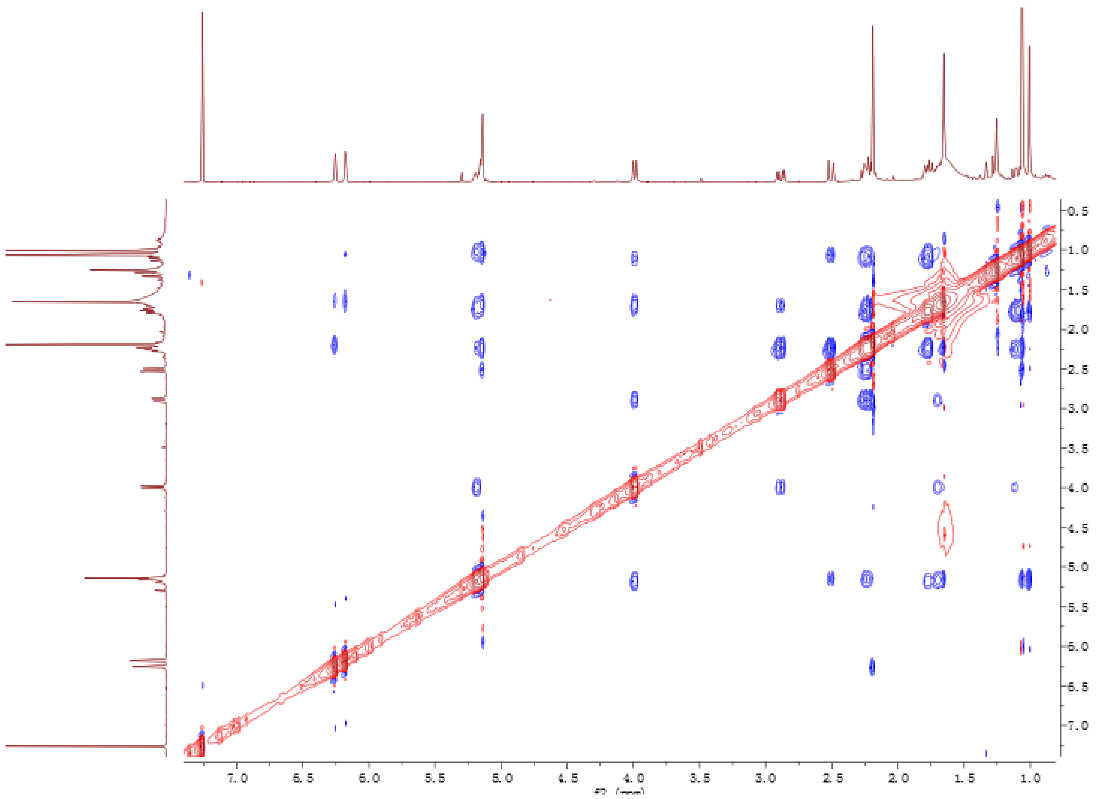

Figure. S13 NOESY spectrum of compound **2**.

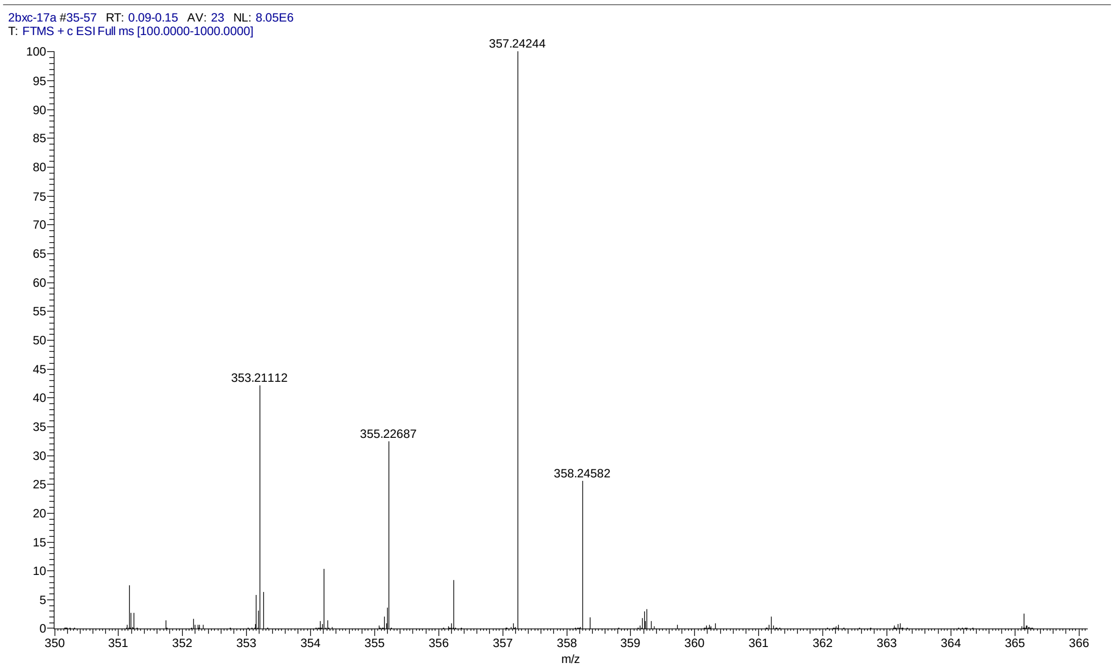

Figure. S14 HRESIMS spectrum of compound **2**.

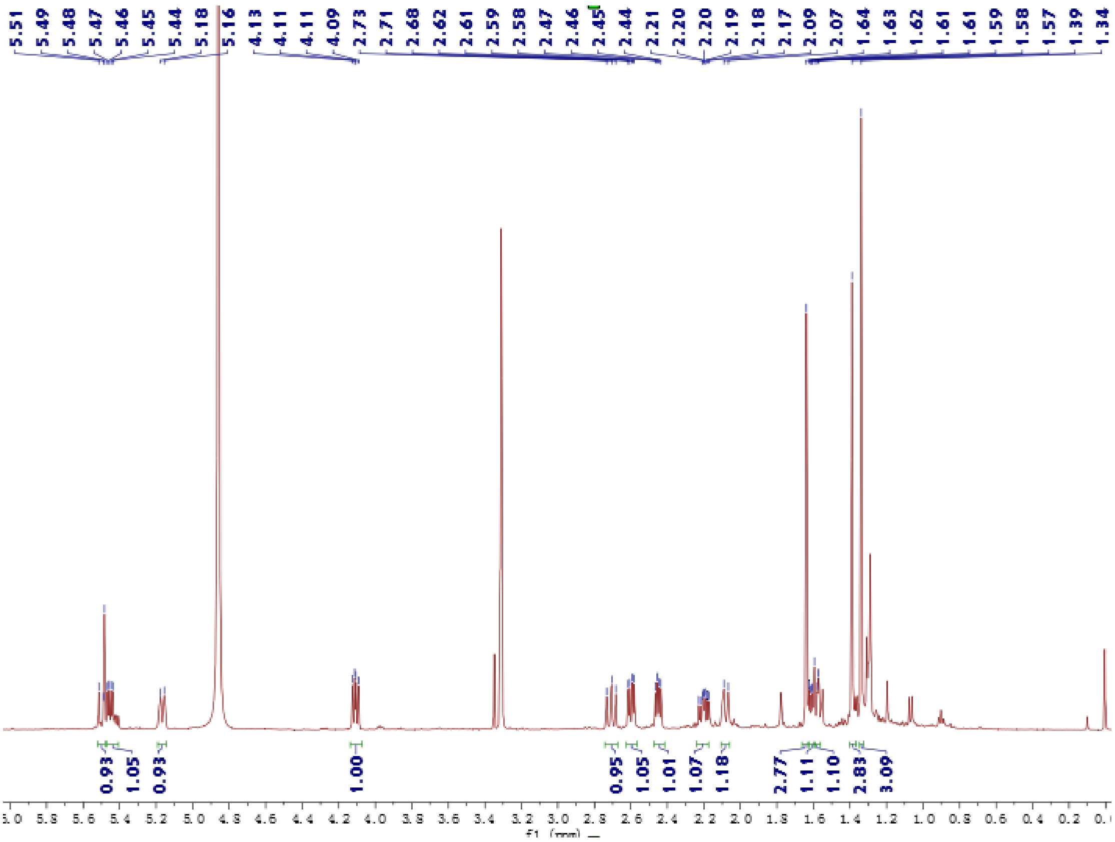

Figure. S15 ^{1}H NMR spectrum of compound **3** (500 MHz, MeOH-d_4).

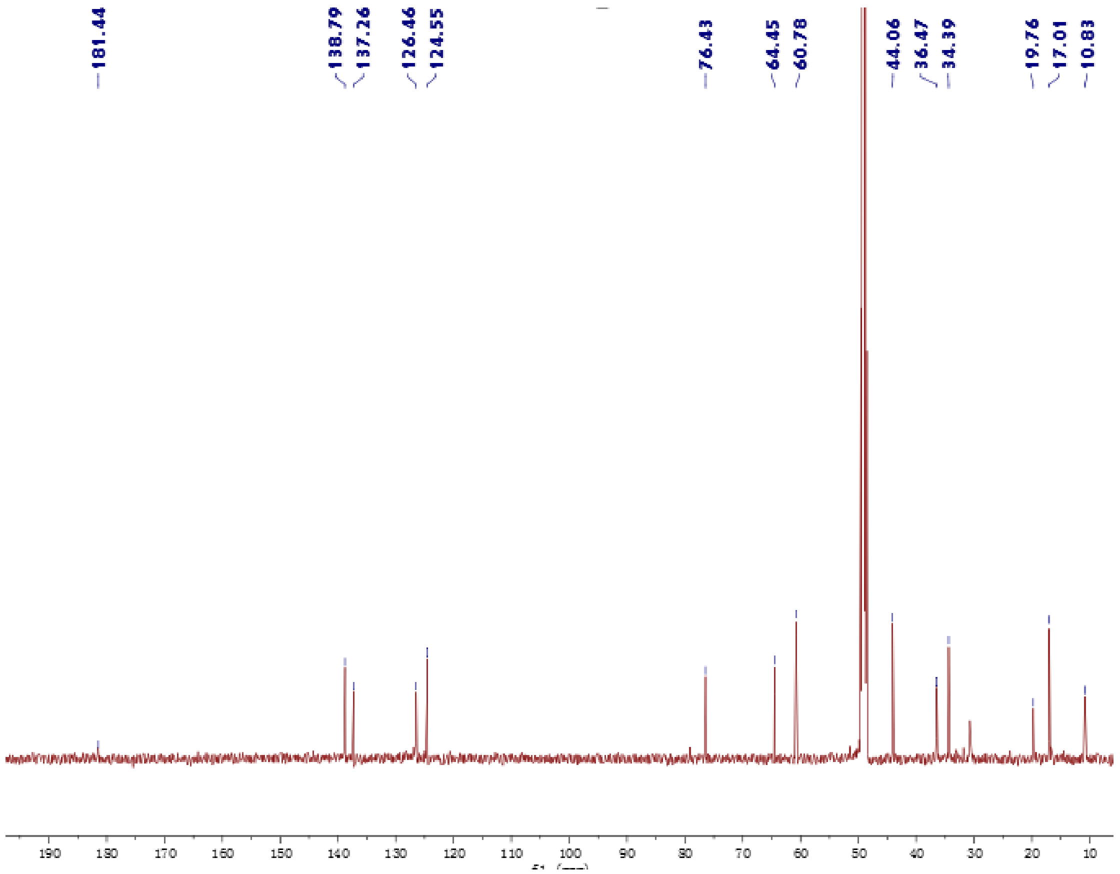

Figure. S16 ^{13}C NMR spectrum of compound **3** (125 MHz, MeOH-d_4).

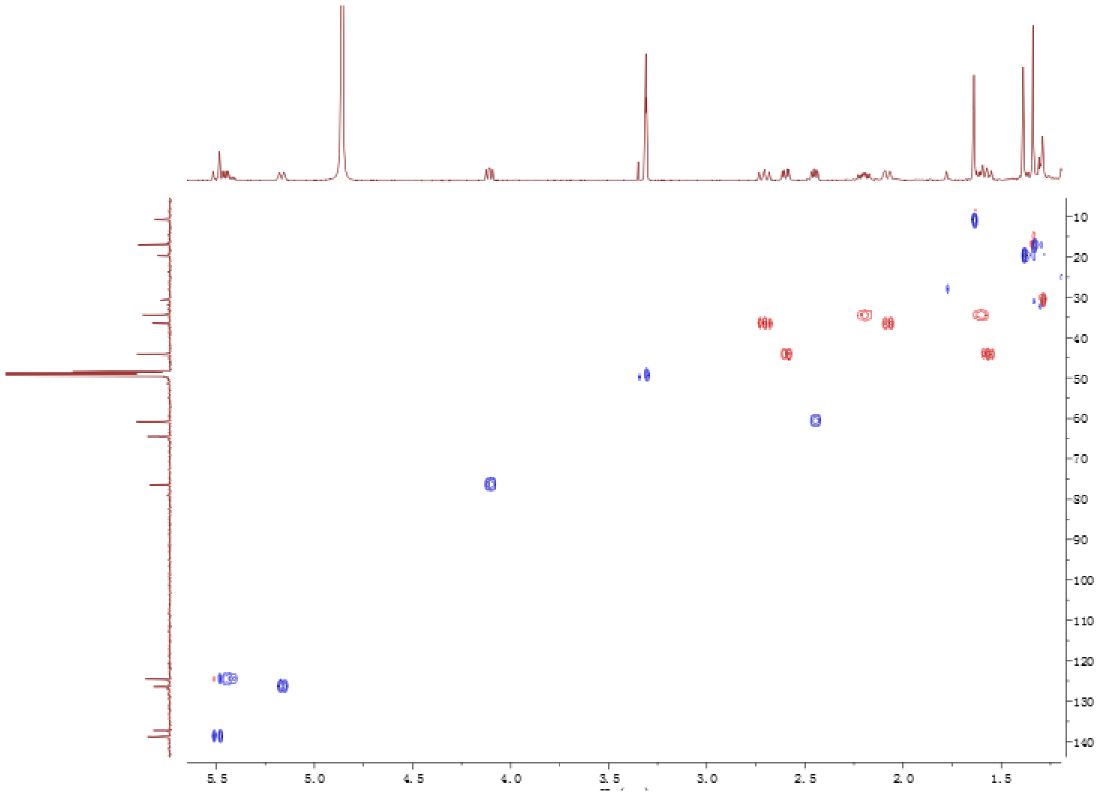

Figure. S17 HSQC spectrum of compound **3**.

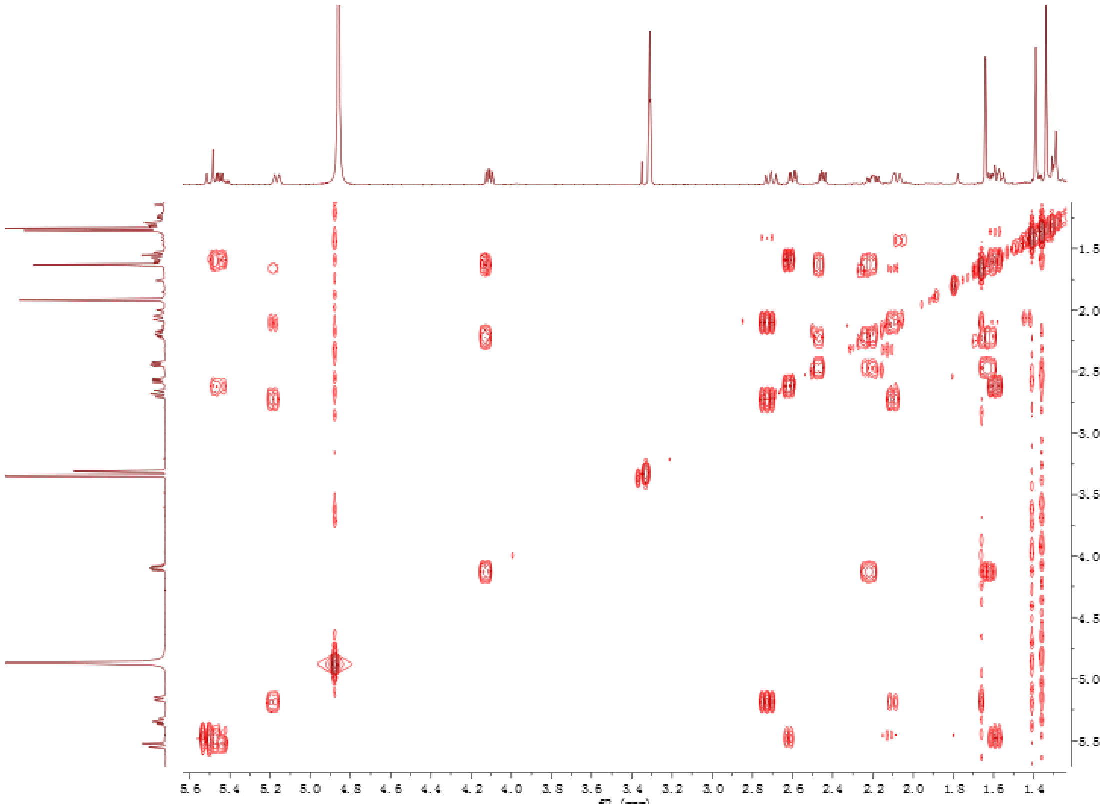

Figure. S18 ¹H-¹H COSY spectrum of compound **3**.

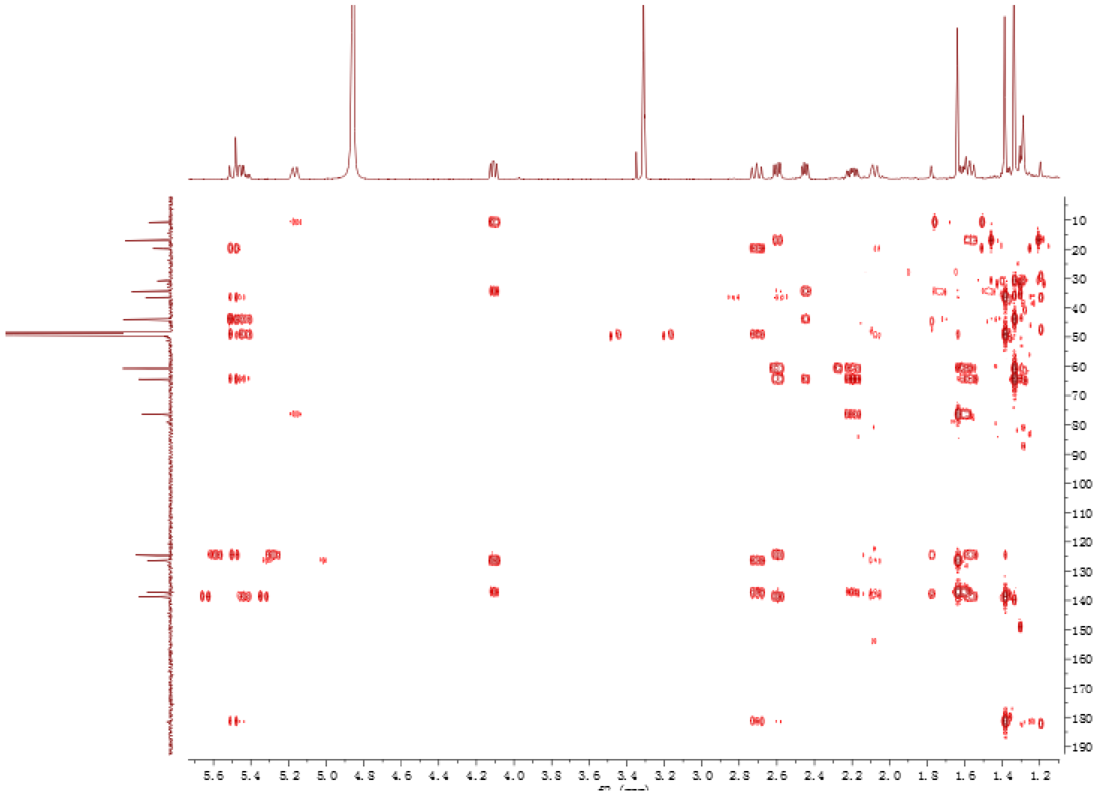

Figure. S19 HMBC spectrum of compound **3**.

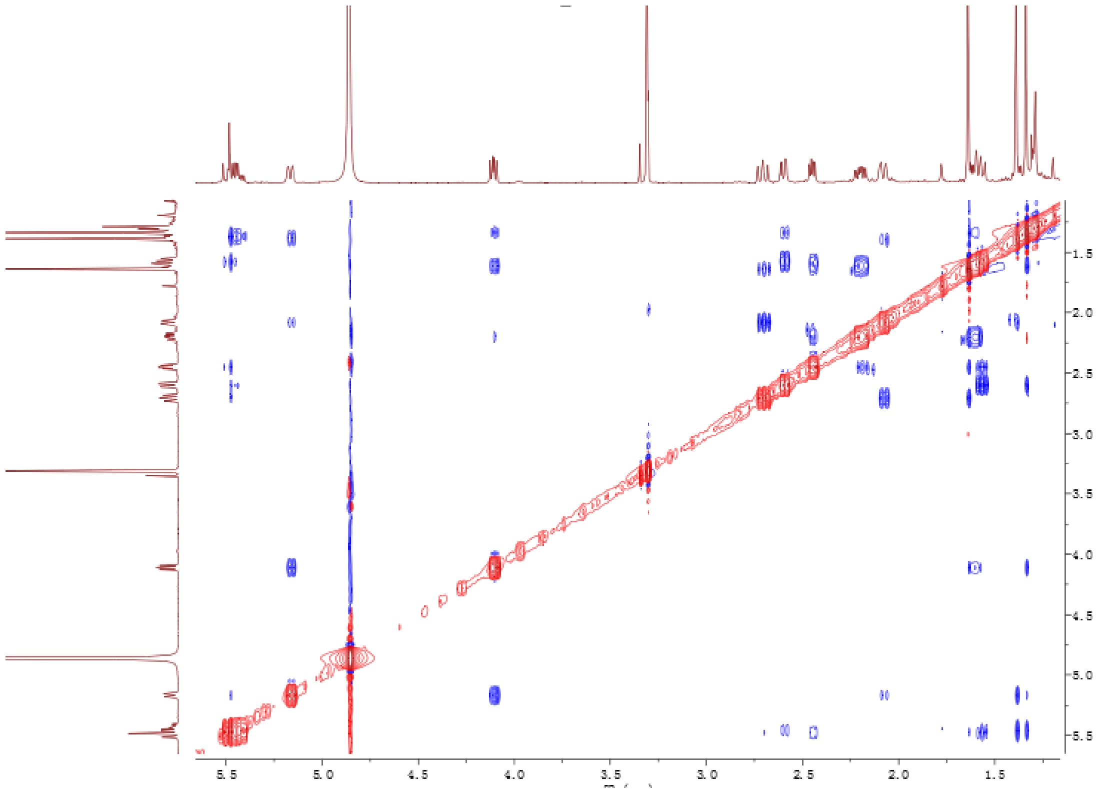

Figure. S20 NOESY spectrum of compound **3**.

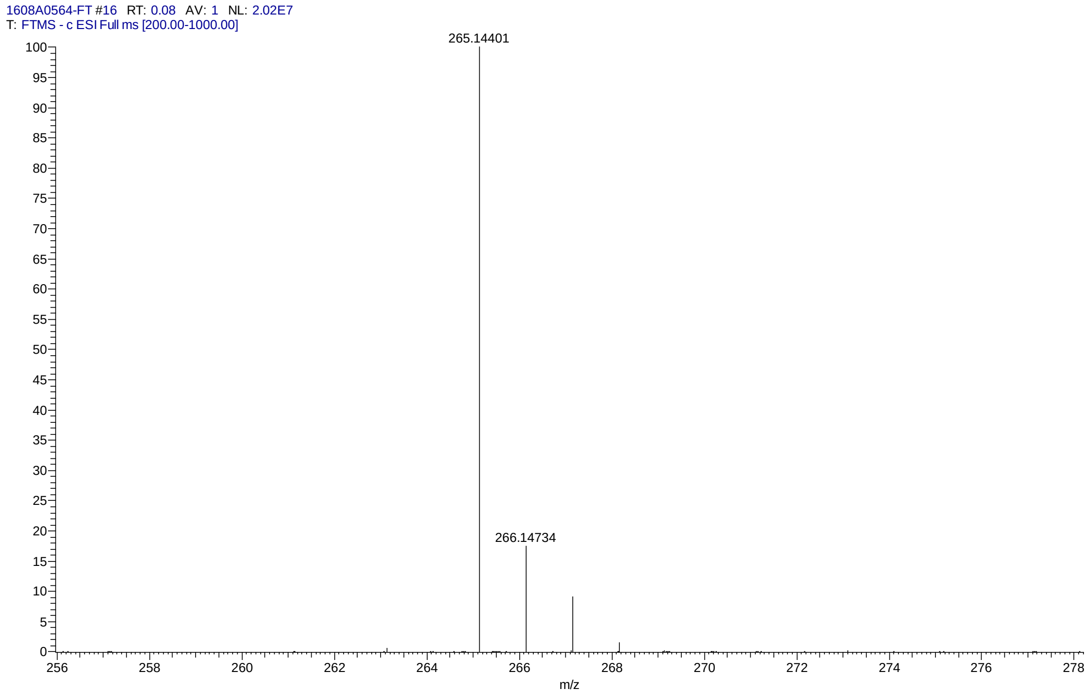

Figure. S21 HRESIMS spectrum of compound **3**.

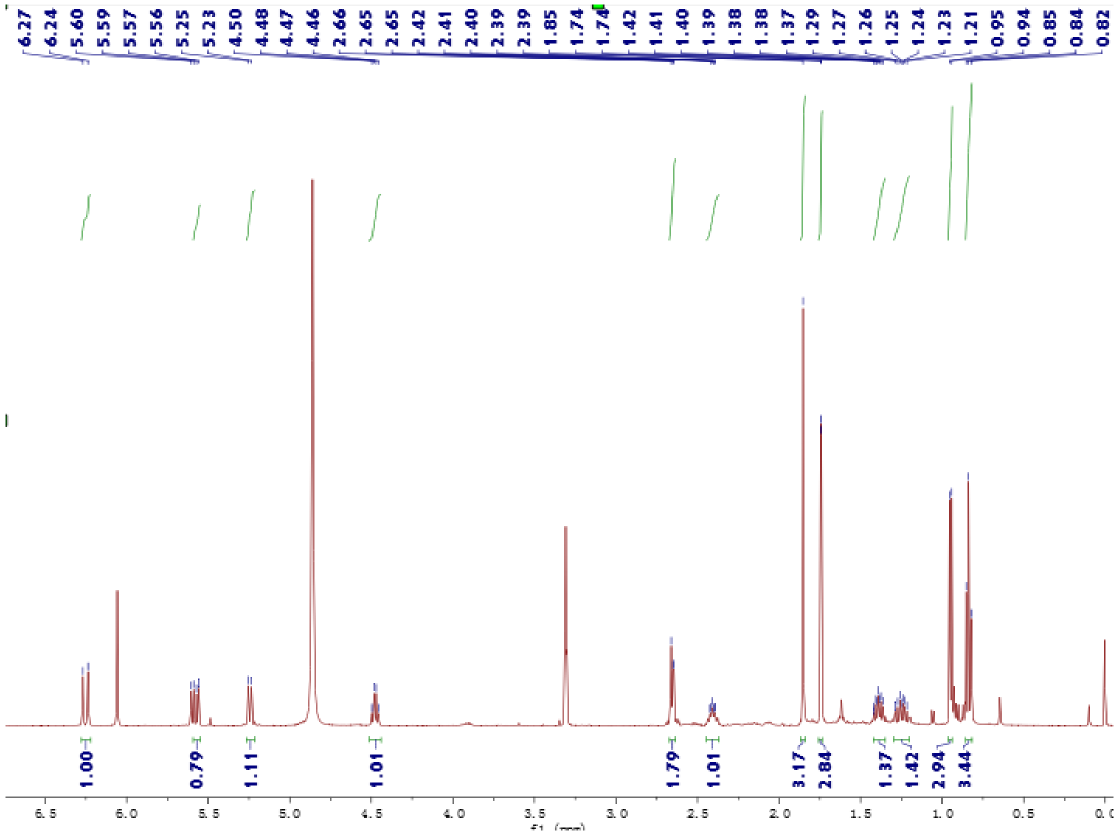

Figure. S22 ^{1}H NMR spectrum of compound **4** (500 MHz, MeOH-*d*$_4$).

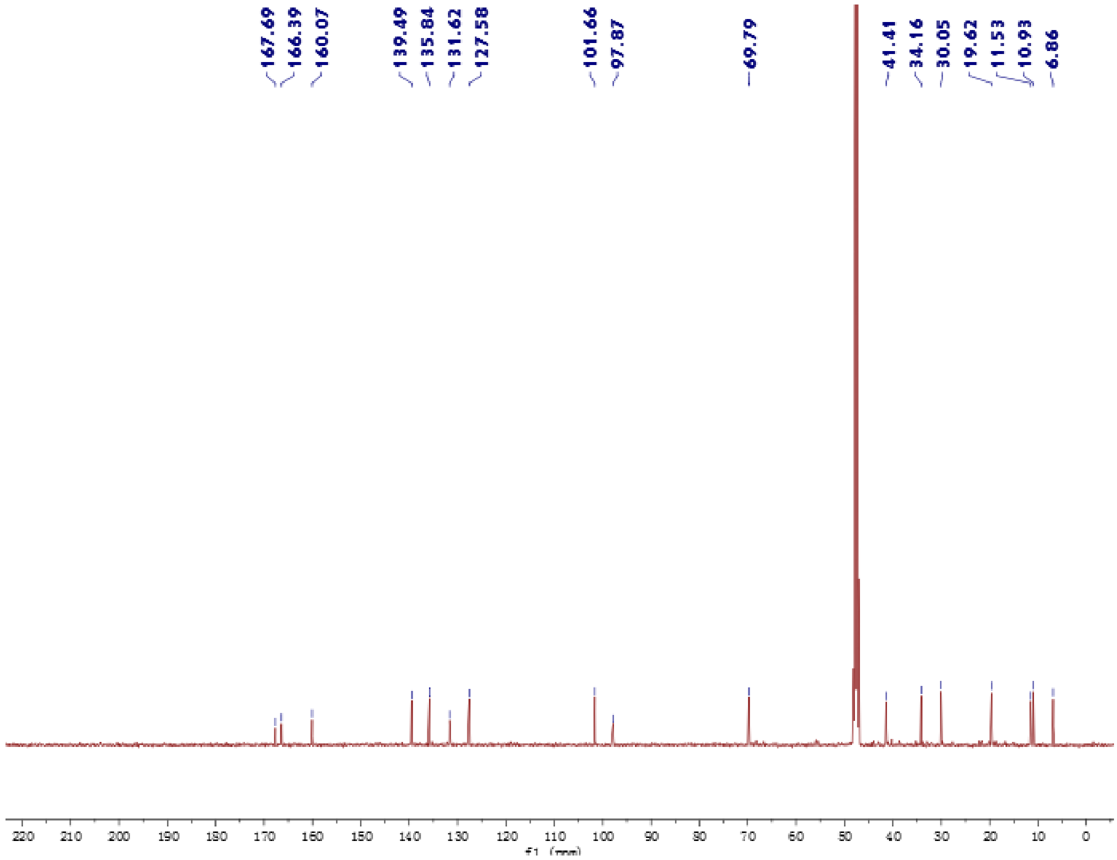

Figure. S23 ^{13}C NMR spectrum of compound **4** (125 MHz, MeOH-*d*$_4$)

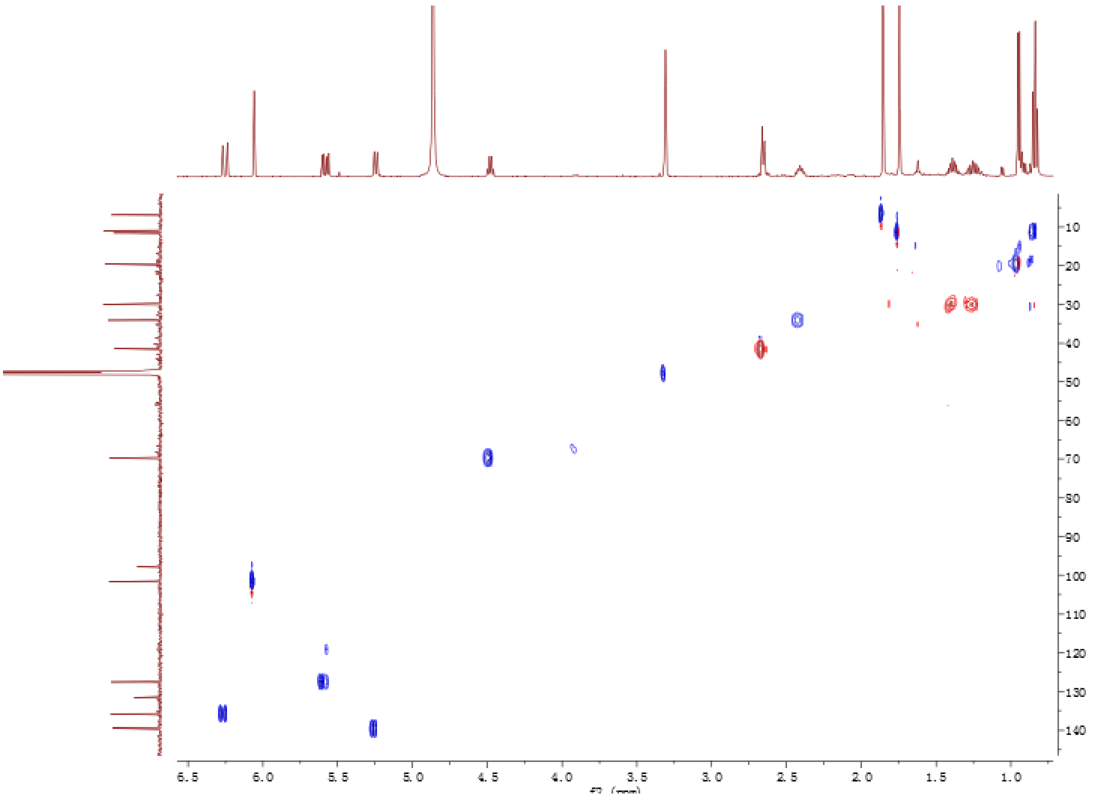

Figure. S24 HSQC spectrum of compound **4**

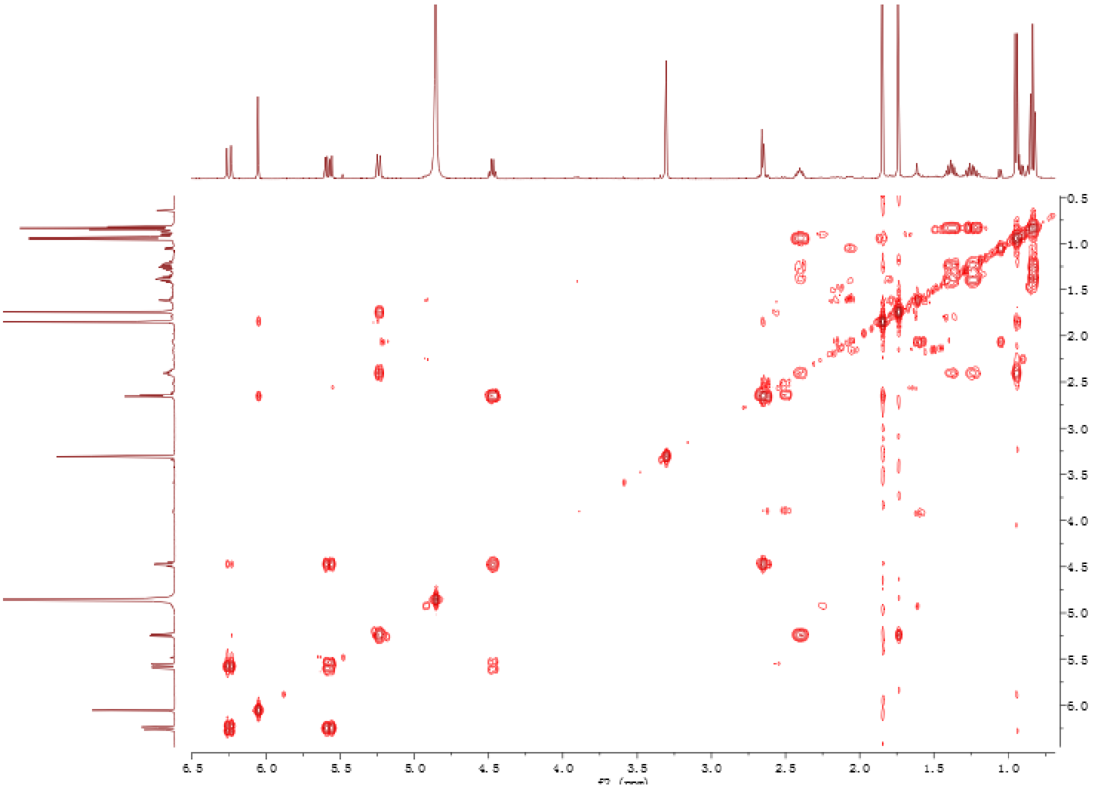

Figure. S25 ^{1}H-^{1}H COSY spectrum of compound **4.**

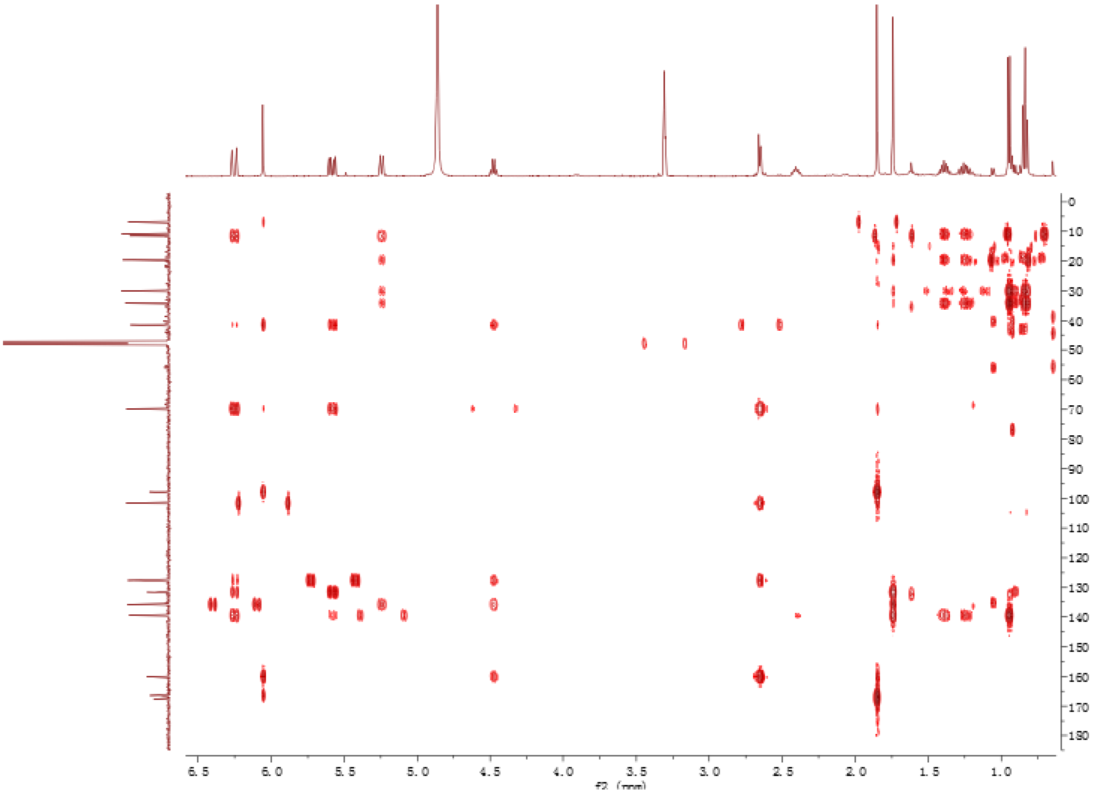

Figure. S26 HMBC spectrum of compound **4**.

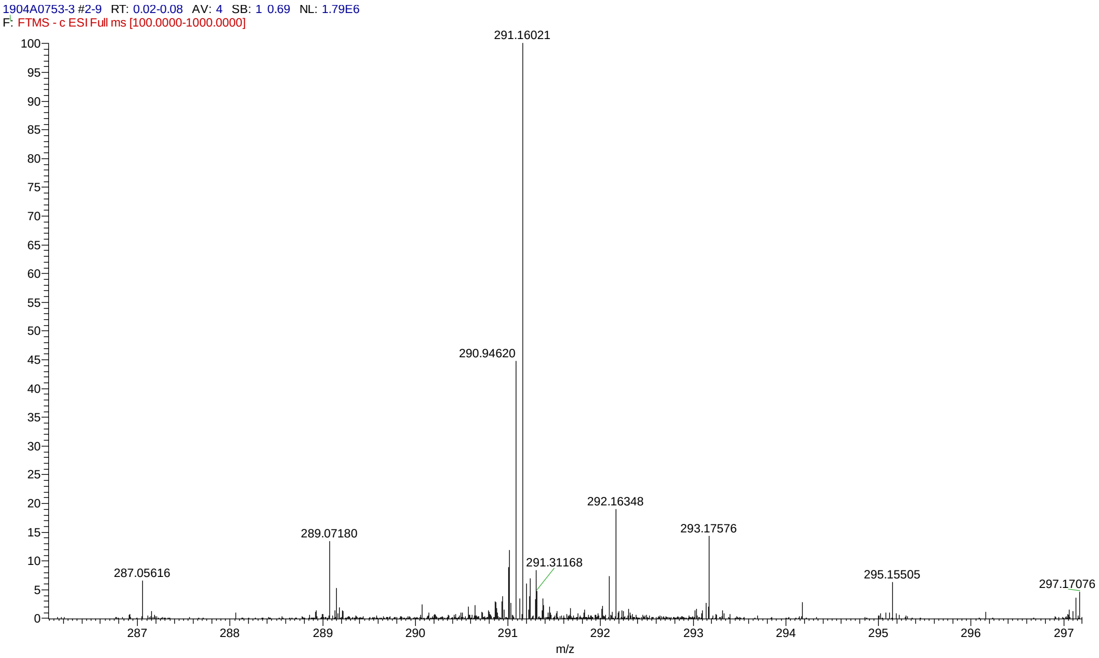

Figure. S27 HRESIMS spectrum of compound **4**.

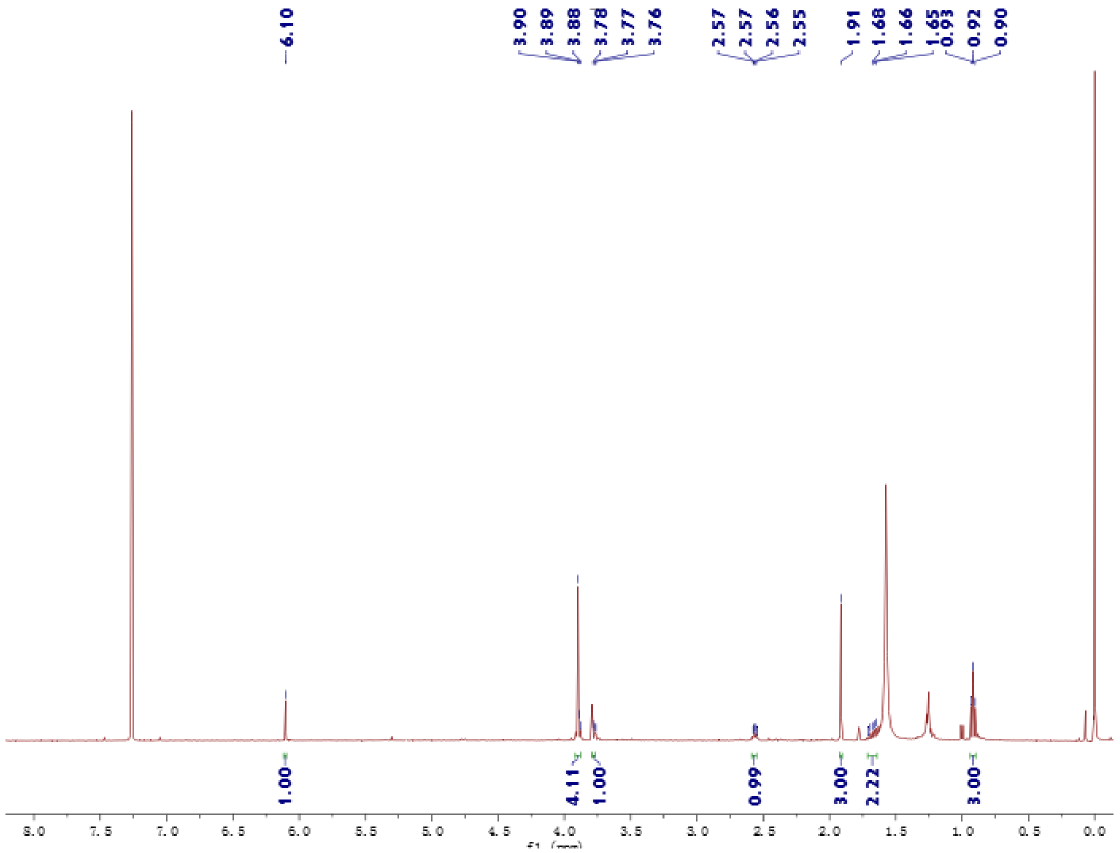

Figure. S28 ^{1}H NMR spectrum of compound **5** (500 MHz, $CDCl_3$)

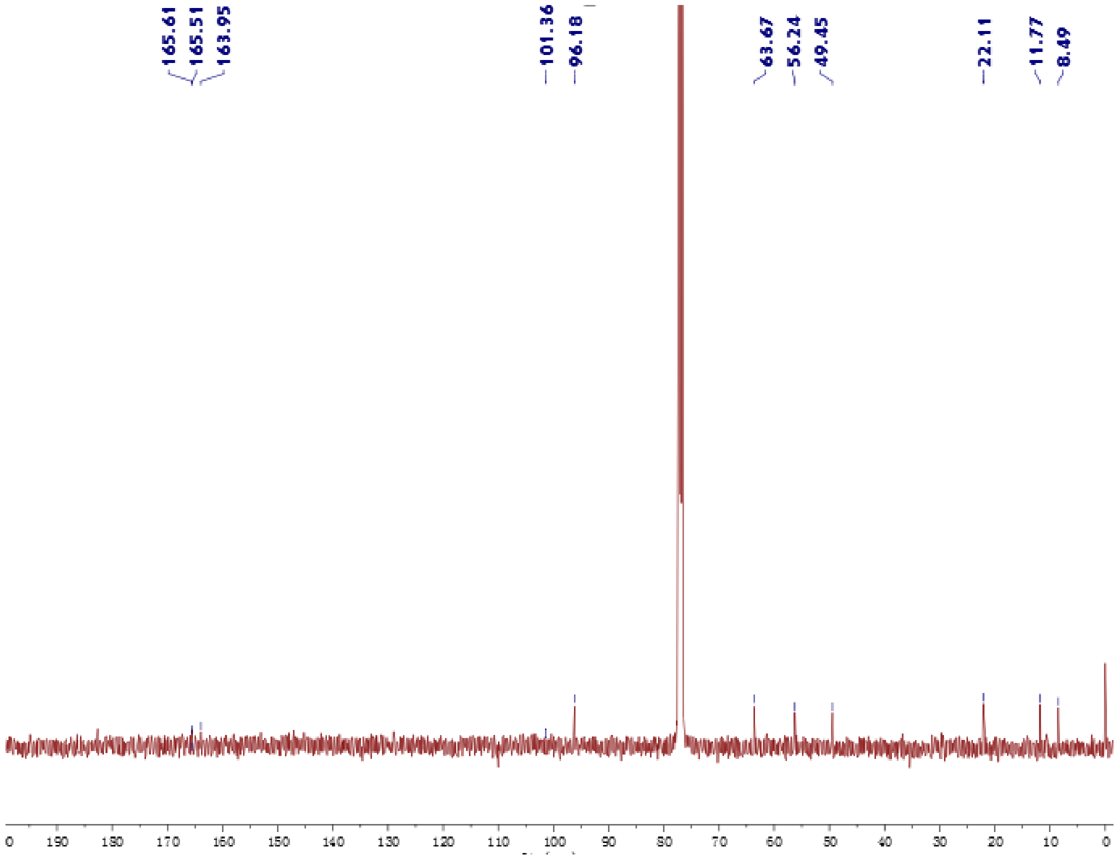

Figure. S29 ^{13}C NMR spectrum of compound **5** (125 MHz, $CDCl_3$)

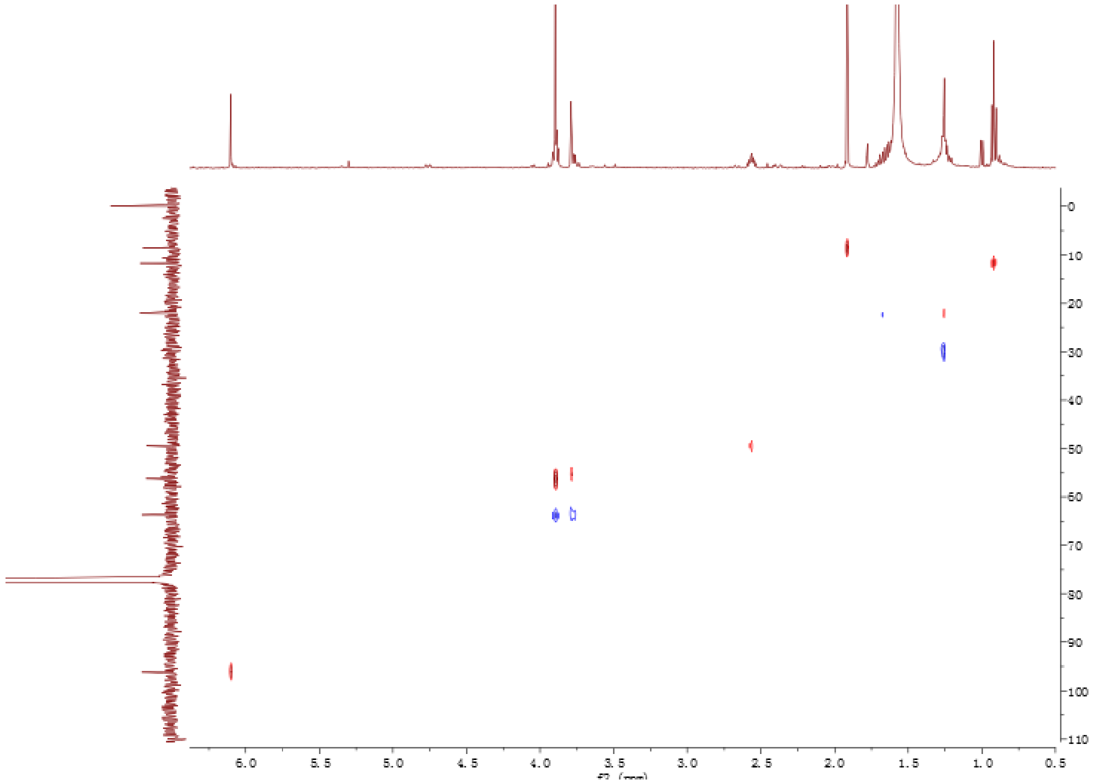

Figure. S30 HSQC spectrum of compound **5**

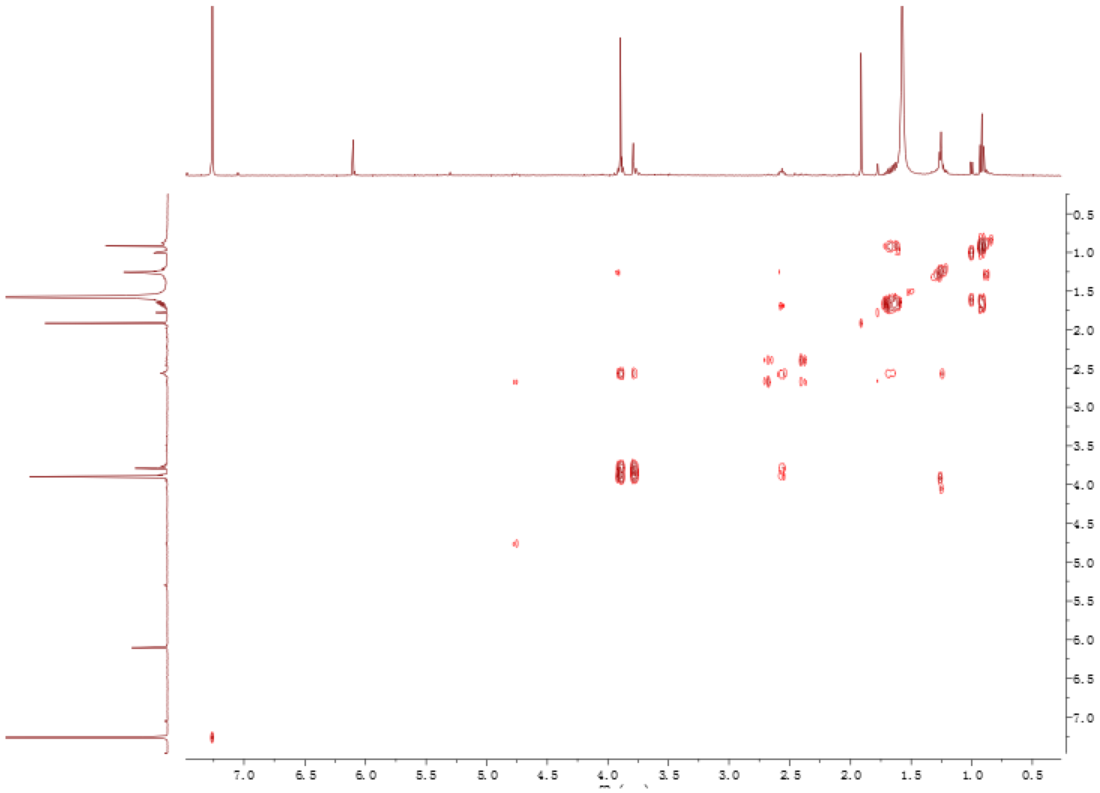

Figure. S31 ^{1}H-^{1}H COSY spectrum of compound **5**

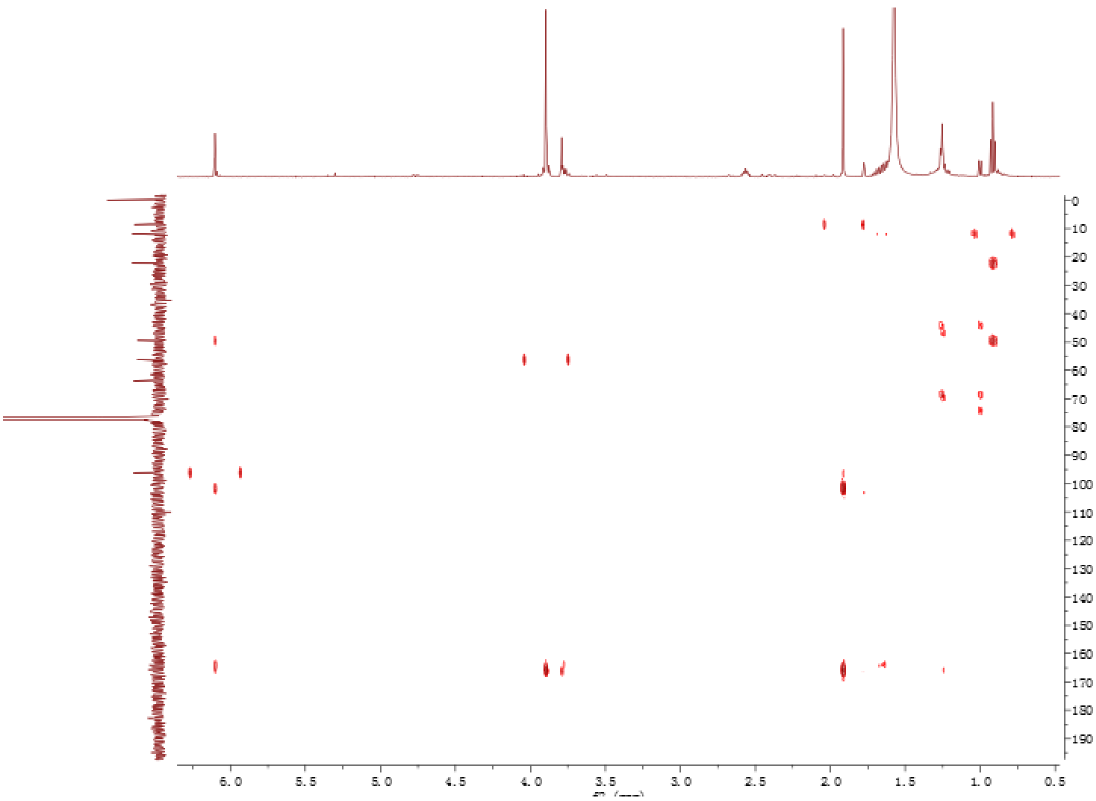

Figure. S32 HMBC spectrum of compound **5**

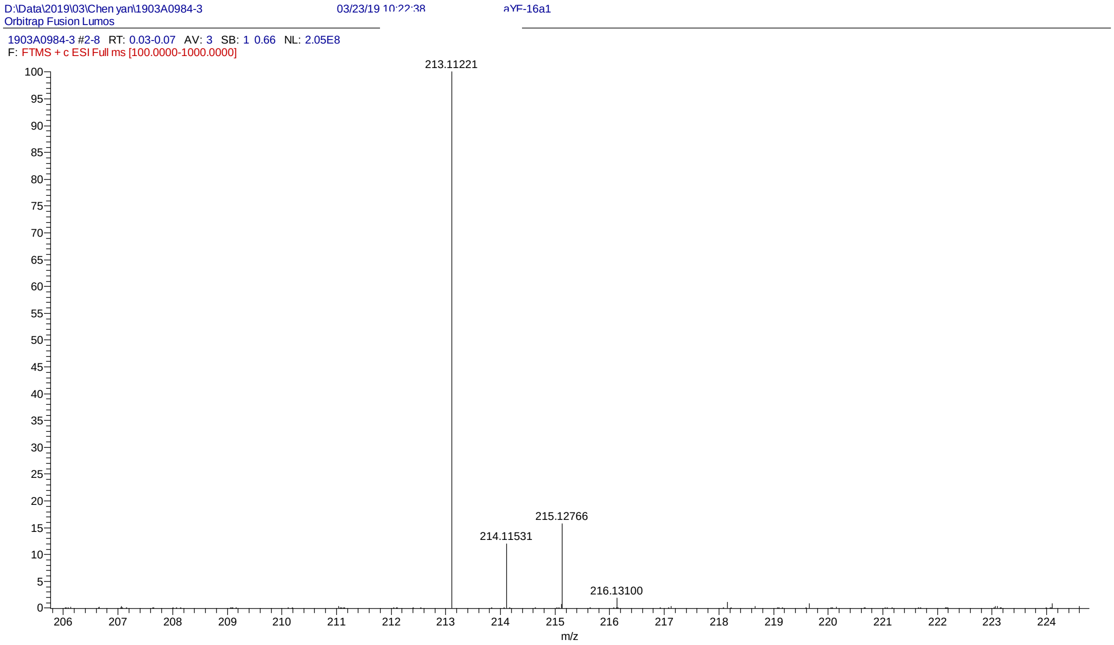

Figure. S33 HRESIMS spectrum of compound **5**

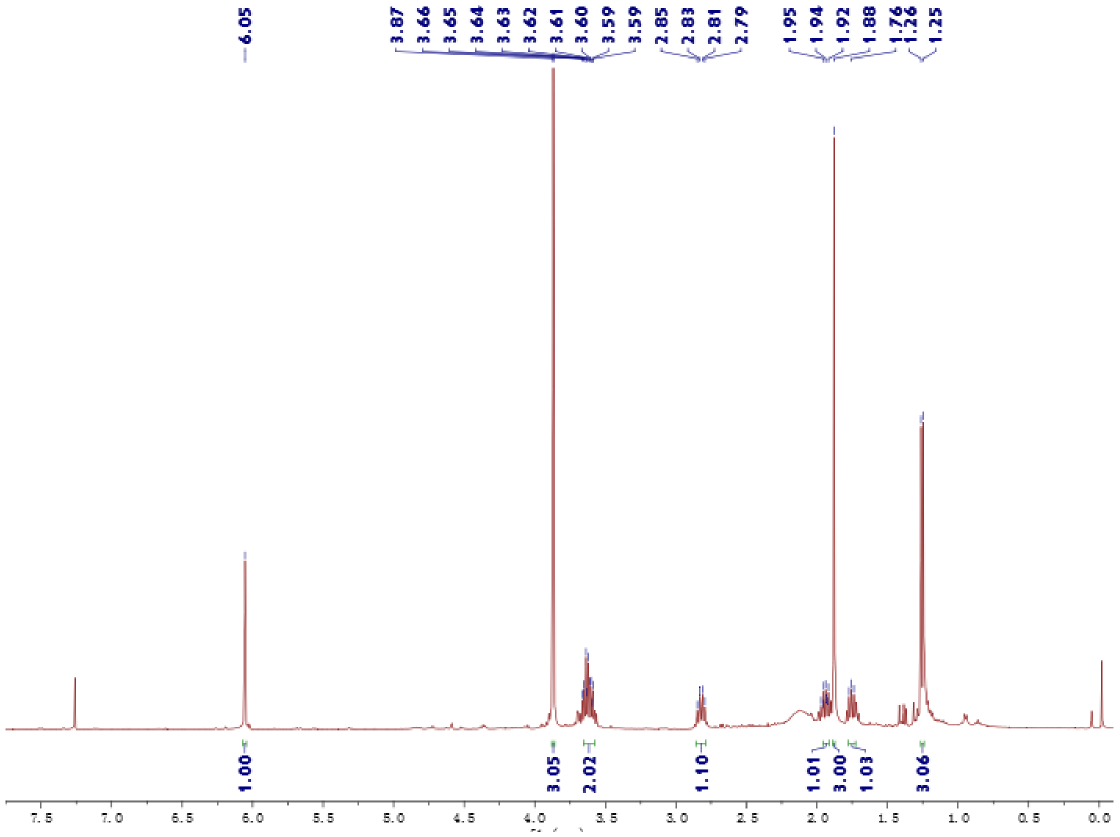

Figure. S34 ^{1}H NMR spectrum of compound **6** (500 MHz, $CDCl_3$)

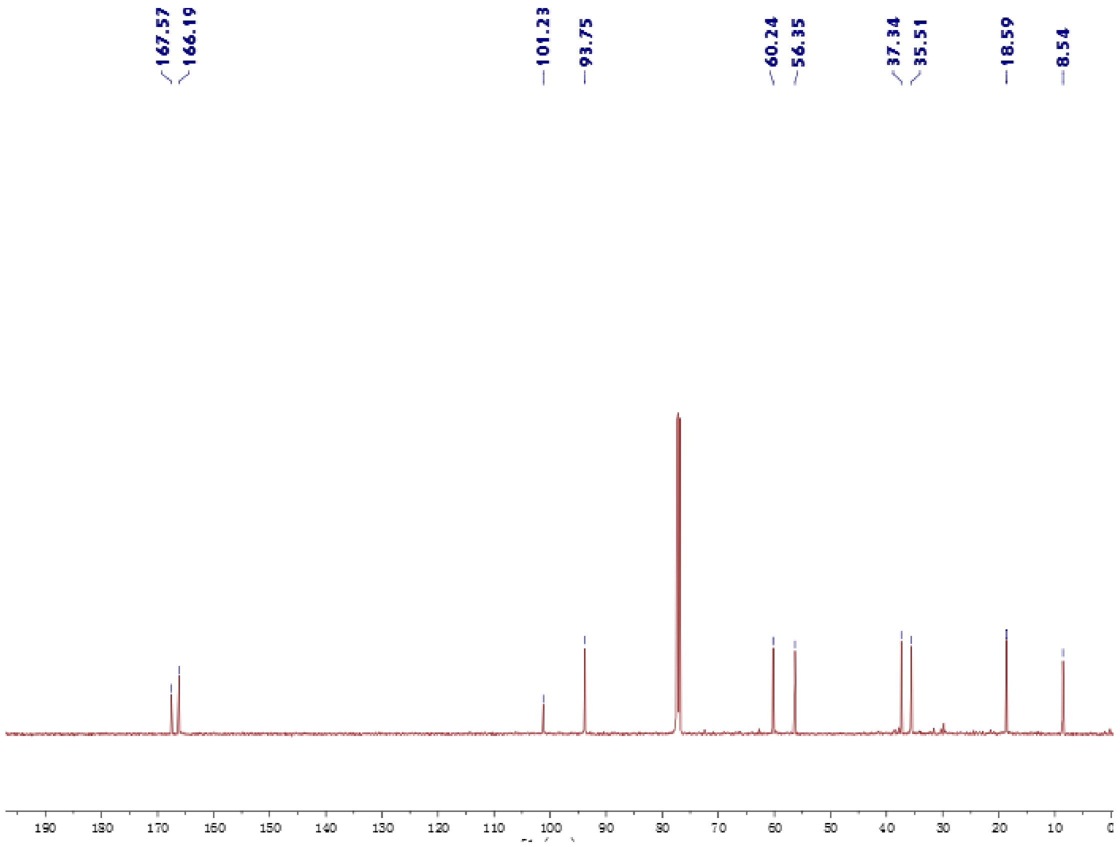

Figure. S35 ^{13}C NMR spectrum of compound **6** (125 MHz, CDCl3)

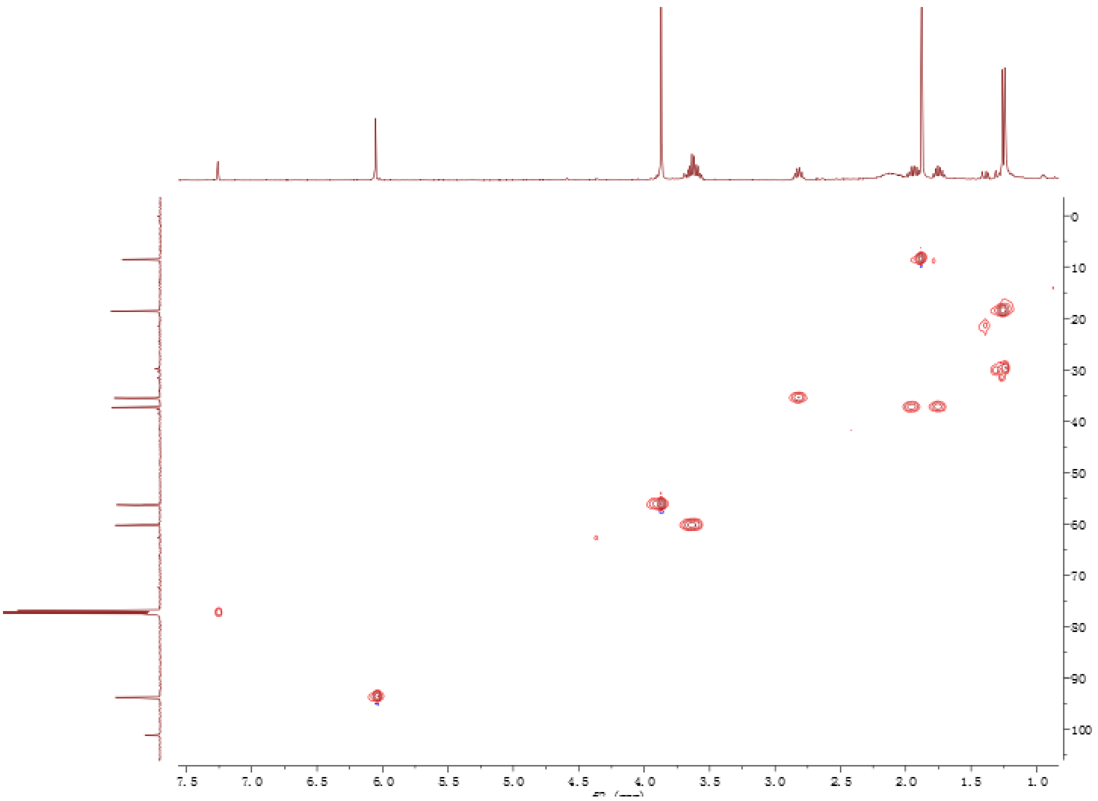

Figure. S36 HSQC spectrum of compound **6**

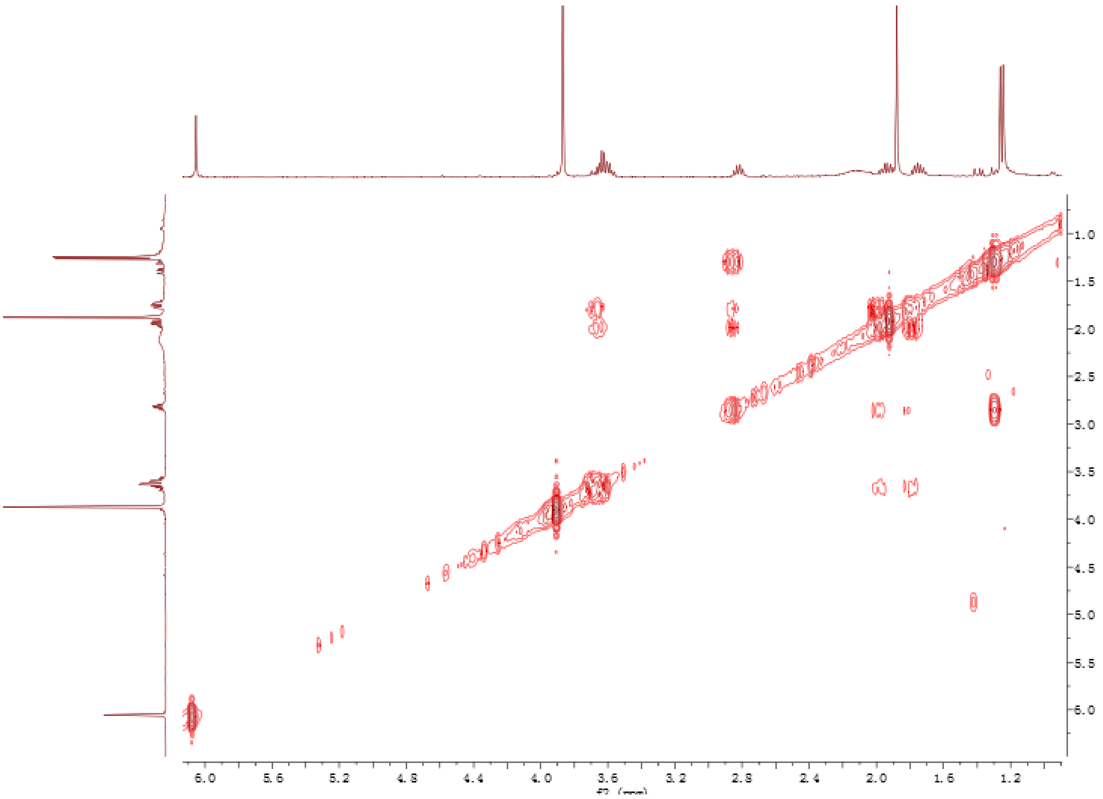

Figure. S37 ^{1}H-^{1}H COSY spectrum of compound **6**

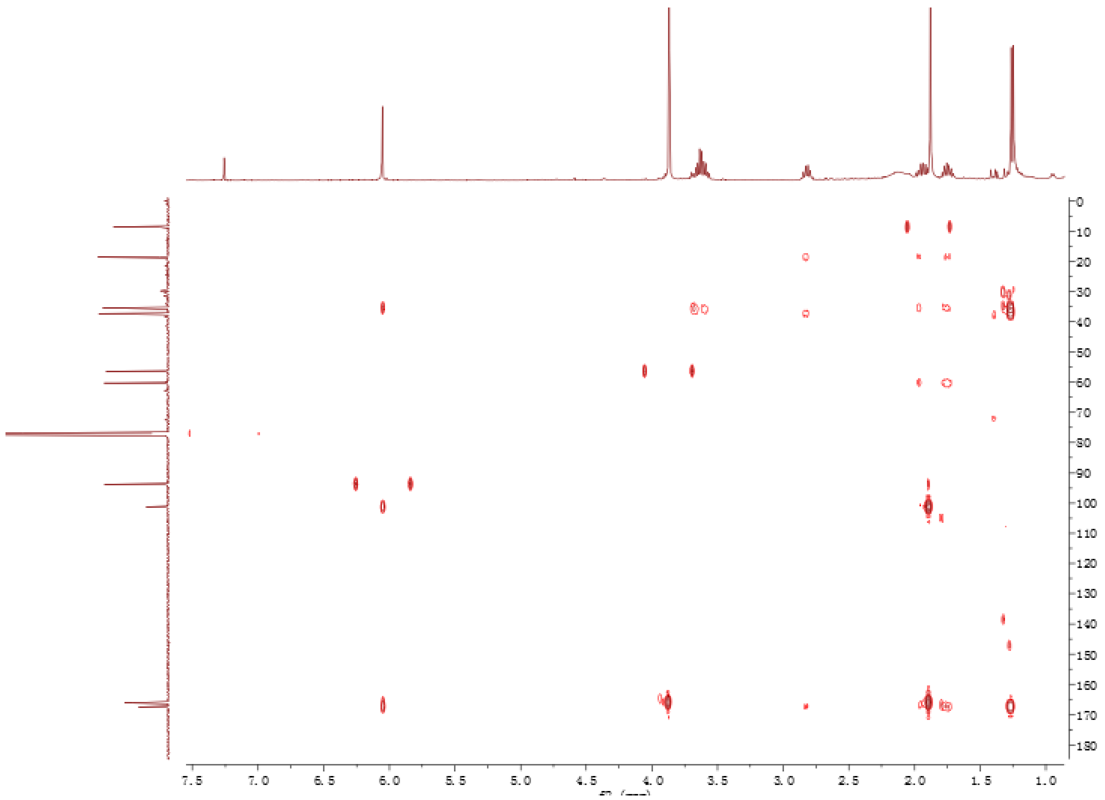

Figure. S38 HMBC spectrum of compound **6**

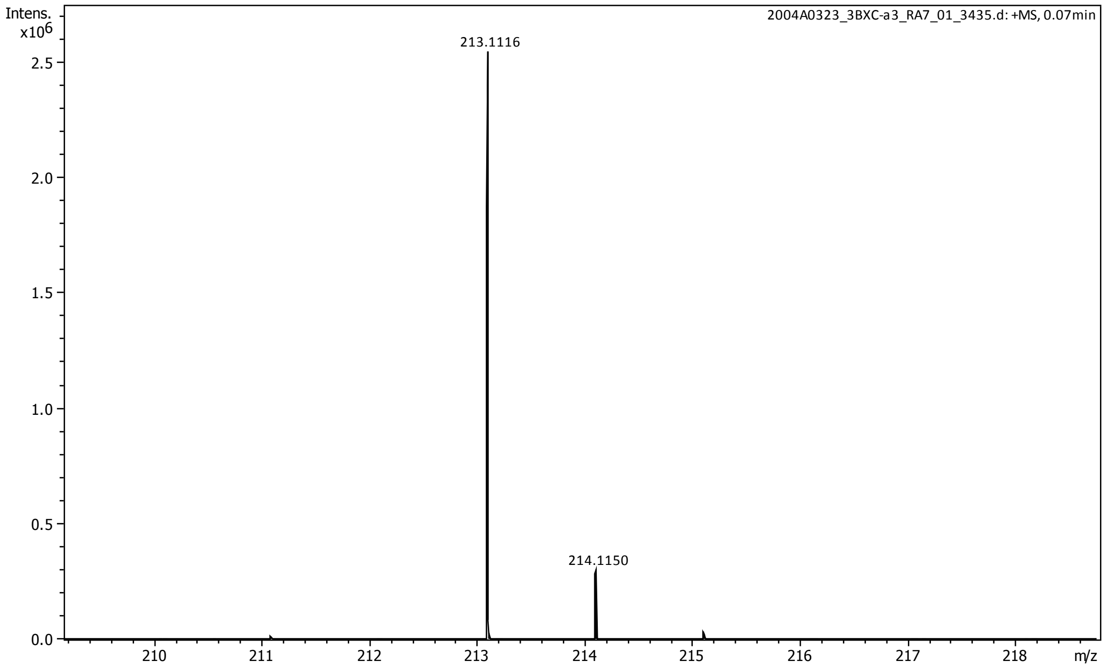

Figure. S39 HRESIMS spectrum of compound **6**

1. The method of anti-inflammatory assay.

Murine macrophage RAW 264.7 cells purchased from Shanghai Institutes for Biological Sciences in DMEM (high glucose) medium supplemented with

10% (v/v) fetal bovine serum, 100 μg mL^{-1} penicillin and streptomycin, and 10 mM HEPES at 37 °C in a 5% CO_2 atmosphere. Cells (1.5×10^5 cells/mL) with LPS (1 μg/mL) and samples (100, 50, 25, 12.5 and 6.25 μM) at 37 °C for 24 h. Briefly, 50 μL of cell culture medium was mixed with 100 μL of Griess reagent, and was incubated at room temperature for 10 min with horizontal shaking. The absorbance was determined at 540 nm wavelength with a microplate reader. N^G-monomethyl-L-arginine (L-NMMA) was used as a positive control, and was purchased from Sigma-Aldrich Co. (CAS number: 53308-83-1). Wells with DMSO were used as a negative control (final DMSO concentration was 0.1 %). The IC_{50} was defined as the concentration of compound that inhibited 50 % NO production relative to the LPS group and was calculated using SPSS 16.0 software. All assays were performed in triplicate.

Cell viability was measured using the conventional MTT assay. RAW 264.7 cells were seeded in 96-well plates at a density of 1.5 × 10^5 cells/mL. After 12 h, the cells were treated with LPS (1 μg/mL) and samples, followed by additional incubation for 24 h at 37 °C. MTT stock solution (2 mg/mL) was added to wells for a total reaction volume of 110 μL. After 4 h incubation, the supernatants were aspirated. The formazan crystals in each well were dissolved in 50 μL of DMSO, and the absorbance was measured using a microplate reader (Multiskan GO, Thermo Scientific, Waltham, MA, USA) at the wavelength of 490 nm.

Table S1. The cell viability of **1-7**.

Compound	**1**	**2**	**3**	**4**	**5**	**6**	**7**
Cell viability (%)	108.371	120.536	122.847	106.536	-	-	112.557

- not tested.
